# Irradiated whole cell *Chlamydia* vaccine confers significant protection in a murine genital tract challenge model

**DOI:** 10.1038/s41541-024-00968-z

**Published:** 2024-11-11

**Authors:** Kieran C. Broder, Vera Y. Matrosova, Rok Tkavc, Elena K. Gaidamakova, Lam Thuy Vi Tran Ho, Andrew N. Macintyre, Anthony Soc, Aissata Diallo, Stephen C. Darnell, Sarah Bash, Michael J. Daly, Ann E. Jerse, George W. Liechti

**Affiliations:** 1 Department of Microbiology and Immunology, Uniformed Services University of the Health Sciences, Bethesda, MD USA; 2 Henry M. Jackson Foundation for the Advancement of Military Medicine, Bethesda, MD USA; 3 Department of Pathology, Uniformed Services University of the Health Sciences, Bethesda, MD USA; 4 Department of Medicine, Duke Human Vaccine Institute, Durham, NC USA; 5 Present Address: Department of Microbiology, Immunology, and Pathology, College of Veterinary Medicine and Biological Sciences, Colorado State University, Ft. Collins, CO USA

**Keywords:** Inactivated vaccines, Bacterial infection

## Abstract

*Chlamydia trachomatis* infections are the most common bacterial STIs globally and can lead to serious morbidity if untreated. Development of a killed, whole-cell vaccine has been stymied by coincident epitope destruction during inactivation. Here, we present a prototype *Chlamydia* vaccine composed of elementary bodies (EBs) from the related mouse pathogen, *Chlamydia muridarum* (*Cm*). EBs inactivated by gamma rays (Ir-*Cm*) in the presence of the antioxidant Mn^2+^-Decapeptide (DEHGTAVMLK) Phosphate (MDP) are protected from epitope damage but not DNA damage. *Cm* EBs gamma-inactivated with MDP retain their structure and provide significant protection in a murine genital tract infection model. Mice vaccinated with Ir-*Cm* (+MDP) exhibited elevated levels of *Cm*-specific IgG and IgA antibodies, reduced bacterial burdens, accelerated clearance, and distinctive cytokine responses compared to unvaccinated controls and animals vaccinated with EBs irradiated without MDP. Preserving EB epitopes with MDP during gamma inactivation offers the potential for a polyvalent, whole-cell vaccine against *C. trachomatis*.

## Introduction

*Chlamydia* are obligate, intracellular bacterial species that are responsible for hundreds of millions of ocular^1^, genital^2,3^, and respiratory^4^ infections each year, representing a major disease burden for the human population^5^. Over 1.6 million cases of *C. trachomatis* were reported in the United States in 2022 alone, with the vast majority of the disease burden falling disproportionately on young African American, Native American, and Native Hawaiian women between the ages of 15 and 24^6^. If left untreated, infections can result in cervicitis, pelvic inflammatory disease (PID), ectopic pregnancy, and infertility. Despite the growing global health priority for vaccines against this ubiquitous human pathogen, few promising candidates for preventing chlamydial infections have emerged in the last 70 years^7^.

Chlamydia’s extracellular, infectious form (elementary body; EB) exhibits a minimal immunostimulatory profile to various innate immune receptors^8^, while the replicative form (reticulate body: RB) effectively evades much of the humoral response to infection as a result of its intracellular niche^9^. Members of the family Chlamydiaceae also utilize a number of mechanisms to suppress the immune response such as altering^10–13^ or reducing the abundance^14–16^ of immunostimulatory structural molecules, secreting effector proteins that directly interfere with host cell apoptosis^17–22^, and suppressing MHC class I and class II expression in antigen-presenting cells^23–25^. Research suggests that a combined humoral and cell-mediated immune response is required for protection and the prevention of disease progression, including the recruitment of macrophages, dendritic cells, natural killer (NK) cells, and CD4+ and CD8+ T cells^26,27^. Development of a robust Th1-driven response involving expression of IL-12 and interferon-gamma (IFNγ), with concomitant downregulation of a Th2 immune response, is effective for driving bacterial clearance^7^, however, TH17-focused responses (in the absence of Th1) have also proven effective^28,29^. Additional cross-sectional analyses of human infections largely agree with experimental animal studies demonstrating that prior infection can grant partial protection to subsequent infections^30^. However, this is not always the case, and is confounded by the fact that many patients who test positive are treated with antibiotics shortly after they are screened, likely before they form a robust protective response.

Previous studies have demonstrated that immunization with either live *C. trachomatis* or the corresponding mouse pathogen, *Chlamydia muridarum*, is capable of conferring measurable protection in animal models^31–35^. However, vaccination strategies utilizing live attenuated bacteria have some fundamental limitations. In addition to the risk of attenuated strains reverting to fully pathogenic organisms and the potential for hybridization with other strains/species^36^, live *C. trachomatis* has been demonstrated to actively suppress a productive CD8+ T cell response, which is essential for efficient bacterial clearance during subsequent infections, in a PD-L1-dependent manner^37^. A few studies have conveyed that immunization with killed organisms is capable of inducing some measurable correlates of protection against *Chlamydia*^31,38–42^, however, the large majority of reports have found that elementary bodies (EBs) killed by heat, chemical agents or radiation largely fail to demonstrate significant protection in animal models^43–52^, as well as in human trials^53–55^. Significantly, antigens from inactivated EBs are often incorrectly targeted to tolerogenic dendritic cells^41^ and stimulation with inactivated EBs (as opposed to live EBs) results in lower levels of peptide loading onto dendritic cells^56^, potentially explaining the reduced levels of protection observed. Several factors may have limited the efficacy of these vaccines, such as the use of different adjuvants, immunization routes, and regimens. The potential damage incurred to surface antigens during the inactivation process may also be an important factor as the efficacy of a whole-cell vaccine is naturally governed by its antigenic potency, and previous inactivation methods (i.e., heat, radiation, and chemicals) may have compromised antigen integrity. More specifically, we propose that the physiochemical approaches applied previously in killing EBs in vaccine development also destroyed multivalent epitopes capable of eliciting strong antibody responses.

Despite some additional data indicating that certain EB antigens may be conditionally deleterious to the induction of a protective immune response^42,57^, present *C. trachomatis* vaccine candidates include one whole-cell inactivated vaccine^41^, two live-attenuated vaccine strains^33,35^, and a number of monovalent synthetic peptides that include a wide assortment of potential chlamydial antigens^58^. Current *C. trachomatis* peptide vaccine candidates that target the pathogen’s major outer membrane protein (MOMP)^59,60^ are promising, but inherently limited by their monovalent nature. In addition to potentially missing other important conformational epitopes for antibody recognition, monovalent peptide approaches may also result in overall reduced vaccine immunogenicity as peptide antigens often do not contain sufficient T-cell epitopes^61^. Finally, unlike the MOMP encoded by other *Chlamydia* species^62^, the MOMP expressed by *C. trachomatis* is highly polymorphic, with 19 serovars currently recognized^63^. Such diversity also extends to chlamydial polymorphic membrane proteins (Pmps)^64,65^, which, like MOMP, are antigenically variable across *C. trachomatis* strains and serovars^66,67^. It is likely that this antigenic variability will limit monovalent peptide strategies against *C. trachomatis* in the long term. The major benefit of a whole-cell *C. trachomatis* vaccine is that no targeted antigen selection strategy is required, as different individuals within a population can respond to many different multivalent antigens present in the vaccine^68^.

To overcome these challenges, we introduce an innovative polyvalent vaccine approach based on whole EBs that leverages the scavenging of reactive oxygen species (ROS) under aqueous γ-irradiation. We utilize a Mn^2+^-Decapeptide (DEHGTAVMLK) Phosphate antioxidant complex, named MDP, to selectively protect chlamydial EB surface proteins from ROS generated by γ-rays while leaving DNA and RNA inside EBs susceptible to destruction by ROS. We show that this technique, derived from the exceptionally radiation-resistant bacterium *Deinococcus radiodurans*, selectively shields chlamydial EB surface proteins from radiation-induced ROS, while effectively inactivating the pathogen’s genome^69,70^. Our proof-of-concept study with *Cm*, a surrogate for *C. trachomatis* in human infections, demonstrates that this approach not only preserves surface epitopes but also maintains the structural integrity of γ-inactivated EBs. Whole *Cm* EBs inactivated by γ-rays in the presence of MDP are highly protective in a murine genital tract infection model, resulting in lower infectious burdens, time-to-clearance, and reduced disease pathology. This methodological improvement in microbe inactivation offers a path towards effective, polyvalent whole-cell vaccines against chlamydial infections.

## Results

### MDP treatment enables the sterilization of chlamydial EBs by γ-irradiation while preserving their structural integrity

**Fig. 1:**
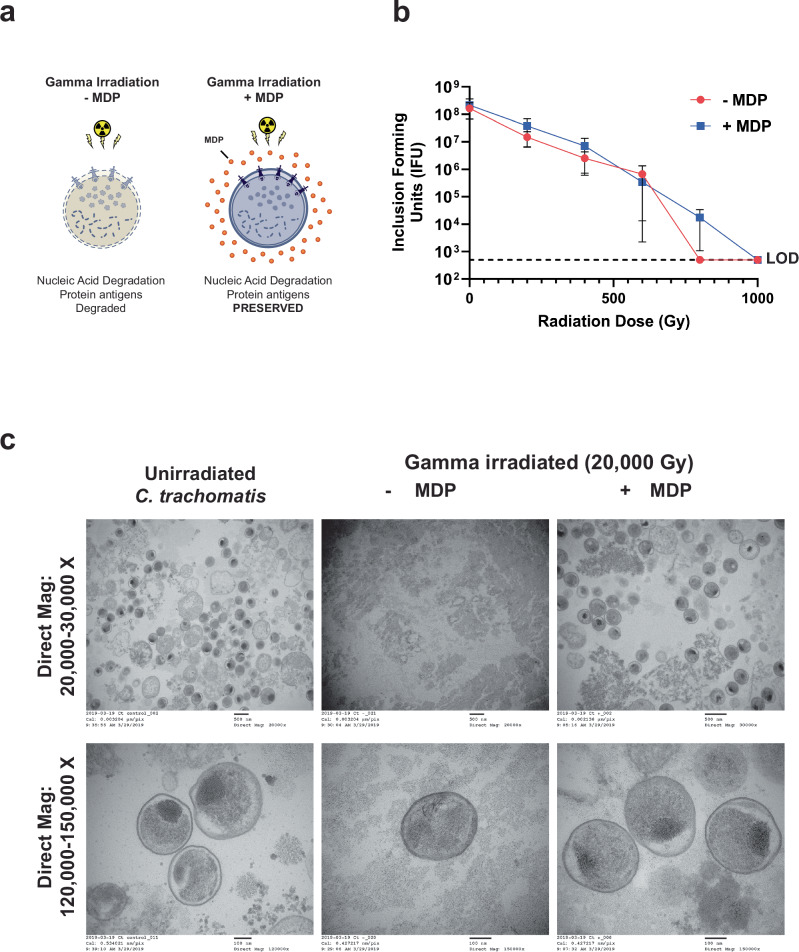
MDP treatment enables the sterilization of chlamydial EBs via γ-irradiation while preserving their structural integrity. **a** Graphical depiction of the protective effects of MDP against ionizing radiation. MDP complex protects proteinaceous components of bacterial outer membranes from ROS-induced damage while allowing for the destruction of the genome. **b** Dose-response γ-irradiation kill curve for *C. trachomatis*. Each data point represents the mean of three biological replicates obtained from three separate experiments (presented individually in Supplementary Fig. 1a). Error bars represent the standard error of the mean. LOD limit of detection. **c** Transmission electron micrographs of *C. trachomatis* EBs (dark spheres) subjected to 20× the LD_100_ of γ-radiation (20 kGy) in the presence or absence of MDP.

The use of MDP during irradiation effectively protects proteins and preserves tertiary epitopes on the surface of cells from degradation by free oxygen radicals, but not the DNA inside of cells (Fig. 1a). *C. trachomatis* EBs were found to be highly susceptible to killing by γ-radiation (Fig. 1b and Supplementary Fig. 1a) with ~1000 Gray (Gy) being sufficient to inactivate ~10^8^ inclusion-forming units (IFU). Our calculated lethal dose (LD) matched a previous study^71^, and was one-tenth the dose used in the only other reported chlamydial vaccination study that utilized γ-radiation^44^. We found that this level of killing was achievable in relatively small volumes of suspended organisms (~100–500 µL), and that MDP did not significantly impact microbial killing (Fig. 1b and Supplementary Fig. 1a). Live EB counts were established via IFU assay, and due to the high numbers of EBs present in our stock preparations, we estimated that a high-confidence limit of detection (LOD) achievable for these assays was ~400 IFU. To examine irradiated chlamydial viability below the LOD of our standard assay, we plated ~2 × 10^8^ IFU of Ir-*Ct* (+MDP) that received a radiation dose of 1 kGy across L2 cell monolayers covering five separate 24 well coverslips, and at 24 h post-infection (hpi) cells were fixed, permeabilized, and immunolabeled with a monoclonal antibody to MOMP. All fields of view were then examined for each coverslip, and no inclusions were observed >2 μm across in samples exposed to 1 kGy. As our major goal was to ultimately test whether γ-irradiated EBs could be used successfully in murine vaccination studies, we also tested the radiation tolerance of the murine chlamydial pathogen *Cm*, and found that 1 kGy was sufficient to inactivate ~10^8^ IFU (Supplementary Fig. 1b).

To demonstrate that MDP is capable of protecting the cellular integrity of EBs undergoing γ-inactivation, we subjected ~10^8^ *C. trachomatis* EBs to 20× our calculated LD (20 kGy) in the presence or absence of MDP, then fixed and imaged the bacteria by transmission electron microscopy. This dose of radiation effectively destroyed almost all of the chlamydial EBs in our control sample (−MDP), with only three EBs recognizable in over 30 fields of view observed (Fig. 1c). By contrast, chlamydial EBs were easily discernable in our MDP-protected sample at numbers comparable to our non-irradiated control. EBs in both non-irradiated and MDP-protected samples appeared to have well-defined inner and outer membranes, and stable foci containing dark-staining chromatin. By contrast, the membrane integrity of EBs in the non-protected, irradiated sample appeared to be severely compromised. Taken together, these results indicate that a radiation dose of 1 kGy is sufficient for inactivating ~10^8^ chlamydial EBs in an aqueous solution, and that MDP prevents the destruction of EBs at higher radiation doses extending to 20 kGy, likely due to its protective effect on the crosslinked, proteinaceous structures that make up the EB surface.

### Live and γ-irradiated EBs have similar internalization rates in tissue culture cells, but exhibit differential TLR signaling

We next set out to assess whether the γ−irradiation procedure adversely affects surface features on the chlamydial outer membrane. The majority of chlamydial outer membrane proteins are thought to play important roles in adherence and host cell invasion^65^, so we tested the ability of *Cm* EBs exposed to 1 kGy of gamma radiation in the presence or absence of MDP to adhere to and enter HeLa cells. Killed EBs (depending on the method of inactivation) are still capable of binding and being internalized by host cells^72,73^, and we found no significant differences in the internalization of live or γ-irradiated *Cm* (data not shown).

To examine the effects of low-dose γ-radiation on TLR-stimulatory ligands (and indirectly assess their susceptibility to damage from our irradiation procedure), we compared the stimulatory potential of four groups of EBs (non-irradiated or irradiated in the presence or absence of MDP) using hTLR2- and hTLR4- reporter human embryonic kidney (HEK) cell lines. The study was done using different multiplicity of infections (MOIs), and TLR signaling was measured at two separate time points (24 h and 48 h) in order to account for the production of new ligands in live samples over the course of the chlamydial developmental cycle (~48 h). We found no significant difference in TLR2- or TLR4-signaling between irradiated and live samples after 24 h (Supplementary Fig. 2a, c). However, we did observe a decrease in TLR2 signaling in irradiated samples at the 48-h time point when lower MOIs were used (Supplementary Fig. 2b), and the presence of MDP did not appear to impact TLR signaling in either live or irradiated samples. These results appear to indicate that the irradiation procedure does not adversely impact the recognition of chlamydial LPS and lipoproteins by their cognizant innate immune receptors, and that upon irradiation chlamydial EBs are no longer capable of producing new, TLR2-stimulating lipoprotein(s).

### Vaccination of mice with the MDP-protected whole-cell vaccine leads to high titers of *Cm*-specific antibodies and neutralizing antibodies compared to EBs irradiated in the absence of MDP

To assess the efficacy of MDP-treated, γ irradiated chlamydial EBs as a potential vaccine, we designed a murine vaccination study utilizing the chlamydial mouse pathogen, *Cm*. Immunizations were carried out utilizing ~10^7^ γ-irradiated (1 kGy) *Cm* EBs (hereafter referred to as ‘Ir-*Cm*’) in the presence or absence of MDP, and mixed with 10 µg of the TLR9-stimulatory adjuvant CpG, which has been used extensively in chlamydial vaccine development to drive Th1-directed immune responses^74–78^. A mucosal prime, systemic boost regimen was used, consisting of an intranasal (i.n.) immunization on day 0 and two subcutaneous (s.c.) boosts on days 14 and 28. Control group animals were immunized with only CpG or PBS on each vaccination day. Vaginal lavage and sera were collected 10 days after the second and third immunization (days 24 and 38, respectively). *Cm*-specific IgG and IgA from individual mice were compared between each group by ELISA (serum) and western blot (serum and vaginal washes).

**Fig. 2:**
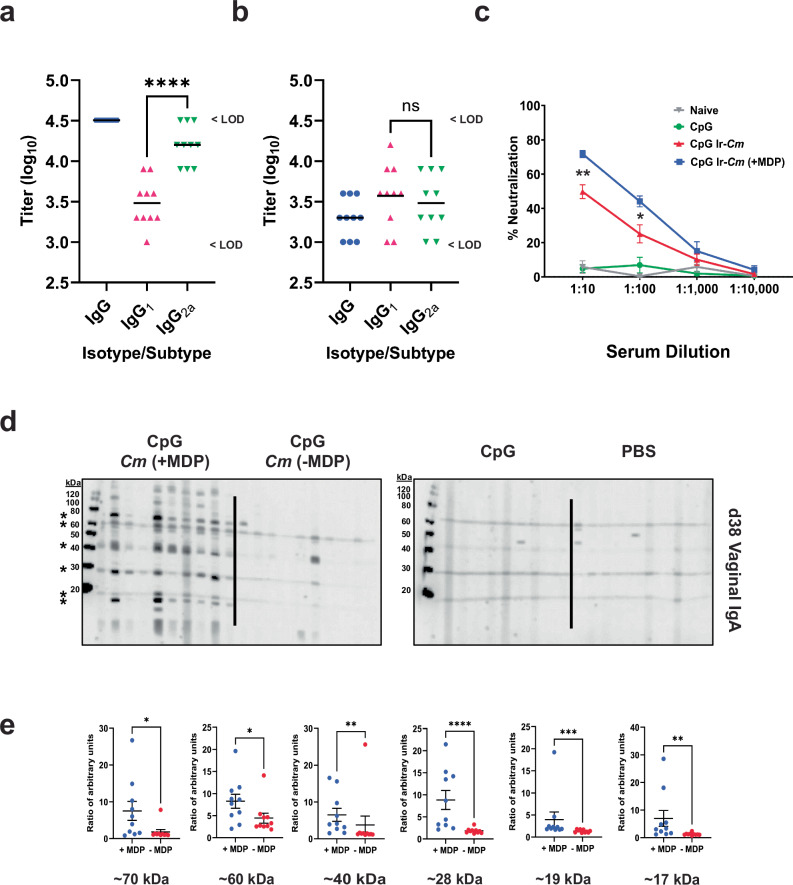
A whole-cell *Cm* vaccine irradiated in the presence of MDP (Ir-*Cm* + MDP) induces *Chlamydia*-specific and functional antibody responses. **a**, **b** ELISA titers of d38 serum from individual mice comprising the Ir-*Cm* (+MDP) (**a**) and Ir-*Cm* (−MDP) (**b**) immunization groups. For the purpose of carrying out a statistical analysis, LOD values were used. Differences between isotypes/subtypes were assessed via one-way ANOVA with Tukey’s test. ****p < 0.0001, ns not significant. **c** Serum neutralization assays comparing pooled serum from the same groups of animals presented in (**a**–**b**). Data points represent four separate neutralization assays from the same pooled sera stocks (collected on d38) using freshly isolated infectious EBs. Error bars represent the standard error of the mean. The 1:10 and 1:100 serum dilutions were compared between the CpG IR-*Cm* and CpG IR-*Cm* + MDP groups via one-way ANOVA with Tukey’s test. **p < 0.01, *p < 0.05. **d** Western blots against fractionated *Cm* EB lysates showing vaginal *Cm*-specific IgA from individual mice vaccinated with *Cm* irradiated (Ir-*Cm*) in the presence or absence of MDP, adjuvant (CpG) alone, or PBS (naïve mice). Vaginal washes were collected on d38; 10 days after the final immunization and tested via WB at a 1:200 dilution. Solid black lines are used to delineate between experimental groups on WBs. **e** Quantitative band intensity analysis for the six most prominent bands from western blots presented in (**d**), (as indicated by asterisks). The mid-line indicates the mean of all data points and the error bars represent the standard error of the mean. Significance was assessed via the Mann–Whitney test. ****p < 0.0001, ***p < 0.001, **p < 0.01, *p < 0.05.

Mice immunized with Ir-*Cm* (+MDP) had high titers of *Cm*-specific IgG (Geo. Mean > 1:32,000) and elevated levels of *Cm*-specific IgG_2a_ (Fig. 2a, Geo. Mean 1:16,000). By comparison, mice immunized with Ir-*Cm* without MDP exhibited more moderate *Cm*-specific IgG levels (Geo. Mean 1:2,000) with no statistically significant difference in their IgG_1_ vs IgG_2a_ isotypes (Fig. 2b). Sera from immunized and control mice were also evaluated by Western Blot against whole cell *Cm* lysates (Supplementary Figs. 3a and 4a, b). As expected, no *Cm*-specific IgG or IgA were detected in sera or vaginal lavages from mice vaccinated with PBS only or the CpG adjuvant control (Fig. 2d; columns 3 and 4 and Supplementary Figs. 3a, b and 4a–c). Serum IgG and IgA from Ir-*Cm* (+MDP)-vaccinated animals recognized more *Cm* proteins than sera from animals immunized with Ir-*Cm* (−MDP) (Supplementary Fig. 4a, b; left panels), with the differences more easily discerned with the lower antibody titers present in serum collected after the second immunization (day 24; Supplementary Fig. 3a).

We also evaluated levels of functional antibodies by assessing their neutralization potential in an EB HeLa cell invasion assay. Neutralization capacity was measured in pooled, heat-inactivated serum collected 10 days after the final immunization (day 38) from naive, adjuvant controlled, Ir-*Cm* vaccinated, and Ir-*Cm* (+MDP) vaccinated mice. In line with our western blot analysis, serum obtained from naïve and CpG-treated mice exhibited no neutralization activity (Fig. 2c). By comparison, serum collected from mice in both our Ir-*Cm* and Ir-*Cm* (+MDP) groups exhibited strong neutralization activity at 1:10 and 1:100 dilutions, with the Ir-*Cm* (+MDP) group consistently displaying ~20% greater neutralization than Ir-*Cm* (*p* < 0.002 and *p* < 0.02, respectively; one-way ANOVA with Tukey’s test).

Mucosal antibody responses were also more easily detectable after the third immunization (day 38), with vaccinated animals displaying robust, *Cm*-specific vaginal IgG (Supplementary Fig. 4c; columns 1 and 2). The difference in *Cm*-specific vaginal IgA at day 38 was even more pronounced (Fig. 2d), with numerous *Cm* proteins recognized by vaginal IgA from all Ir-*Cm* (+MDP) animals compared to Ir-*Cm* (−MDP) animals. A quantitative band intensity analysis for the six most predominant, *Cm*-specific protein bands recognized by IgA present in vaginal washes from immunized animals indicated significant differences (three out of six bands on d24 and six out of six bands on d38) existed in antigen recognition between the Ir-*Cm* and Ir-*Cm* (+MDP) groups (Supplementary Fig. 3c and Fig. 2e).

### Splenocytes from animals vaccinated with MDP-protected Ir-*Cm* exhibit a unique cytokine expression profile upon restimulation

Cell-mediated responses are an important correlate of immunity for obligate intracellular pathogens. We therefore sought to examine cytokine and chemokine responses in restimulated splenocytes from vaccinated animals. *Cm* infection is known to confer a consistent and measurable level of cell-mediated protection against subsequent challenge^41^. We took advantage of this by adding a fifth ‘vaccination’ group to our study (for use as a positive control) where naïve mice were immunized via the intrauterine (i.u.) route with ~10^5^ IFU of live *Cm* on day 10 and allowed to naturally clear the infections (assessed by IFU counts from vaginal swabs). Splenocytes were collected from all vaccinated and control mice on day 38 (ten days after the final immunization). Cells were either left unstimulated or re-stimulated with *Cm* EBs that were γ-irradiated in the presence or absence of MDP for three days. The supernatants were then collected and submitted for cytokine secretion analysis using a multiplex (Luminex) assay.

**Fig. 3:**
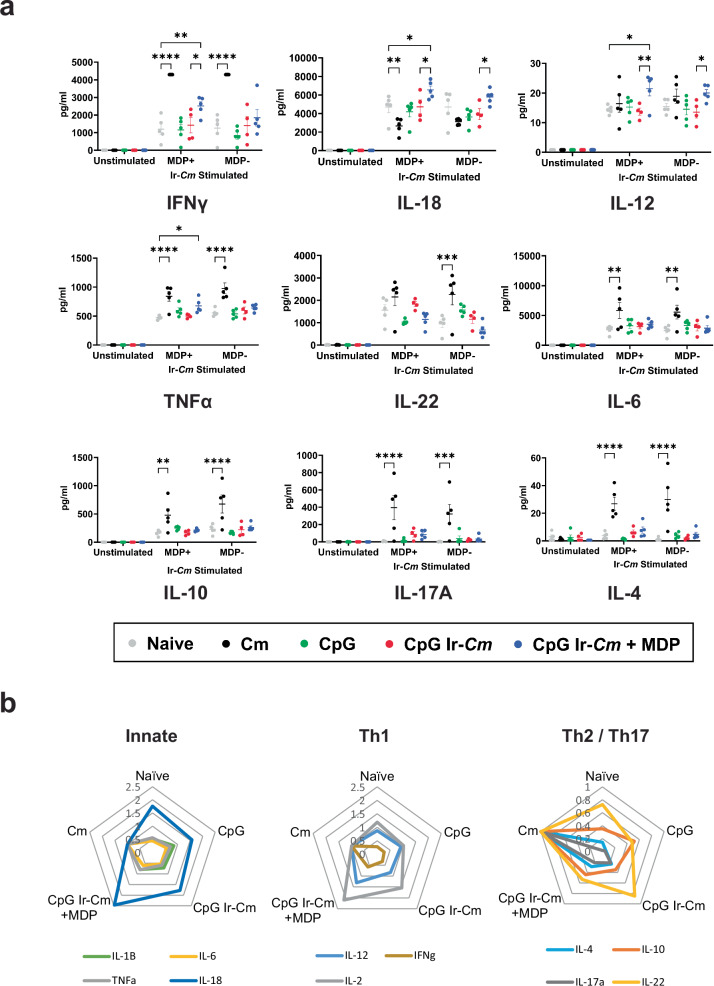
Ir-*Cm* induces *Chlamydia*-specific cell-mediated immunity in an MDP-dependent manner. Spleens were obtained from naïve animals, animals previously infected with *Cm*, mice immunized with adjuvant (CpG), or *Cm* EBs irradiated in the presence (+) or absence (−) of MDP. Each group consists of spleens from five mice (exception, CpG Ir-*Cm* group, *n* = 4) that were stimulated with *Cm* irradiated in the presence or absence of MDP (MDP+/MDP−, respectively). Cytokine induction was assessed using the Luminex 17-Plex assay. **a** Levels of nine cytokines produced by splenocytes restimulated with Ir-*Cm* (+/− MDP). Differences in individual cytokine levels (between the control and vaccine groups) that reached statistical significance are presented by asterisks. Error bars represent the standard error of the mean. Groups were compared utilizing two-way ANOVA with multiple comparisons. ****p < 0.0001, ***p < 0.001, **p < 0.01, *p < 0.05. **b** Spider plots for all cytokines/conditions examined from splenocytes restimulated with Ir-*Cm* (+MDP). All cytokine levels were normalized to the *Cm* pre-exposed group (*Cm*) and individual cytokines were split into three plots based on functional category. Plots using exact cytokine values produced from splenocytes restimulated with either Ir-*Cm* or Ir-*Cm* + MDP are provided in Supplementary Fig. 6.

We were able to examine the levels of 11 different cytokines from our re-stimulated splenocytes. Splenocytes did not produce any significant cytokines when cultured in the absence of antigen (Fig. 3a, unstimulated), providing a steady baseline for further assessment. A Principle Components Analysis (PCA) was conducted to reduce the dimensionality of our data, and we found that while significant overlap existed in our naïve, adjuvant, and Ir-*Cm* groups, both our pre-exposed *Cm* group and our Ir-*Cm* (+MDP) group formed their own unique clusters (Supplementary Fig. 5). When each cytokine was examined individually, we found that cells obtained from mice pre-exposed to live *Cm* prior to challenge exhibited significantly heightened levels of IFNγ, TNFα, IL-6, IL-10, IL-17A, IL-1β, and IL-4 (Fig. 3a and Supplementary Fig. 6b). For the vast majority of cytokines examined (IFNγ, TNFα, IL-6, IL-10, IL-17A, IL-1β, and IL-4), the cells responded equally well to stimulation with EBs that were irradiated in the presence or absence of MDP, with the notable exception of IL-22, which was only enhanced when stimulated with *Cm* irradiated in the absence of MDP. Interestingly, splenocytes harvested from mice immunized with Ir-*Cm* (+MDP) demonstrated a different cytokine profile (Fig. 3a, b). These vaccinated splenocytes showed enhanced levels of IFNγ and TNFα, but also IL-18, IL-12, and IL-2 (Fig. 3 and Supplementary Fig. 6). In the case of IFNγ, IL-18, and IL-12, these responses were only present when splenocytes were stimulated with EBs irradiated in the presence of MDP: no comparable enhancement was present when EBs irradiated in the absence of MDP were used. In contrast, IL-2 was enhanced when stimulating EBs were irradiated in the presence or absence of MDP. Splenocytes from animals given adjuvant alone or vaccinated with EBs irradiated in the absence of MDP did not produce any cytokines above the levels observed in those acquired from naïve animals. Collectively, the splenocyte data demonstrate that the Ir-*Cm* (+MDP) immunized group exhibited a robust Th1 cytokine response and significantly reduced Th2 and Th17 cytokine responses when compared to splenocytes obtained from animals pre-exposed to live *Cm* (Fig. 3b). In addition, these data also indicate that the Ir-*Cm* (+MDP) vaccine candidate results in a unique splenocyte cytokine profile upon restimulation that differs significantly from all other control groups.

### Vaccination with MDP-protected, γ-irradiated *Cm* EBs decreases time to clearance, bacterial burden, and incidence/severity of hydrosalpinx in a murine genital tract challenge model

**Fig. 4:**
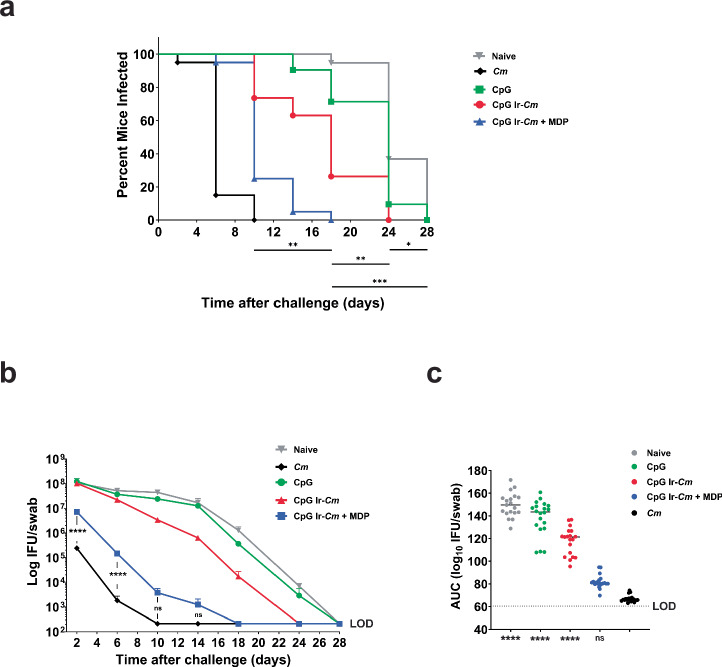
Ir-*Cm* (+MDP) enhances bacterial clearance from the genital tract in a murine vaccination/challenge model. Mice vaccinated with Ir-*Cm* (+/− MDP), administered adjuvant (CpG) alone, PBS (Naïve), or pre-exposed to *Cm* infection (*Cm*) were challenged with live *Cm* 1 month after the final immunization. % infected over time (**a**), average number of IFU/mL vaginal swab suspension over time (**b**), and overall infectious burden calculated from an Area Under the Curve (AUC) analysis (**c**) are shown. Combined data from two separate experiments are shown, each of which tested 10 BALB/C mice/group. (data from each separate experiment are presented in Supplementary Fig. 8). Kaplan–Meier curves were analyzed by the Mantel–Cox test to compare differences in the percentage of culture-positive mice over time. The complete results of the Mantel–Cox test are presented in Supplementary Table 1. Differences in the average (Log10) IFU recovered were assessed utilizing a two-way ANOVA with multiple comparisons. For AUC, groups were compared utilizing a Kruskal–Wallis test with multiple comparisons, and statistical significance readouts are shown for comparisons to the group of mice that was pre-exposed to *Cm* (positive control). ****p < 0.0001, ***p < 0.001, **p < 0.01, *p < 0.05, ns not significant, LOD limit of detection.

To assess the vaccine efficacy of MDP-treated, γ-irradiated chlamydial EBs, we designed an immunization challenge study using the murine *Cm* infection model. Our study design consisted of five groups of ten mice. We challenged mice that were immunized with CpG–Ir-*Cm*, Cp Ir-*Cm* (+MDP), CpG alone, or PBS using the i.n. prime, s.c. boost immunization regimen (*n* = 10 per group; Supplementary Fig. 7). A fifth group of mice was inoculated i.u. with live *Cm* on day 28, which is 28 days before the challenge, as a positive control, as prior infection with *Cm* confers enhanced protection against subsequent infections in mice, as measured by lower bacterial burdens and a faster time to clearance. Seven days prior to the challenge, progesterone was administered to all of the mice. Mice from all groups were then vaginally challenged with ~10^5^ IFU of *Cm*. Venous blood and vaginal lavages were collected 10 days after the 2nd and 3rd immunization and at the end of the challenge phase for assessment of serum and vaginal antibody responses. To assess time-to-clearance and bacterial burden over the course of infection, vaginal lavages were collected every four days, beginning two days post-challenge. Lavages were serially diluted, and used to infect cell monolayers, enabling the calculation of IFU and thus an estimation of bacterial burden over time for each animal. Two independent vaccination/challenge experiments were conducted and the results were similar. Combined data are shown in Fig. 4 and results from each individual challenge experiment are shown in Supplementary Fig. 8.

We found that 95% of unvaccinated animals remained culture positive for *Cm* at 18 days post-challenge compared to no animals that had been previously exposed to live *Cm* or vaccinated with Ir-*Cm* (+MDP) (Fig. 4a). Durations of infection were markedly lower in mice immunized with live *Cm* or with CpG Ir-*Cm* (+MDP), with ~80% of mice clearing infections by days 6 and 10, respectively (Fig. 4a). Mice that were immunized with CpG Ir-*Cm* showed an intermediate level of protection compared to the unimmunized group with 63% infected on day 14 post-immunization. The difference in the duration of infection for the two Ir-*Cm* immunized groups was also significant (*p* < 0.0001). When bacterial burden was assessed over the time following challenge, a stark decrease was evident in the Ir-*Cm* (+MDP) vaccinated group (*p* < 0.0001) and the pre-exposed *Cm*, positive control group (*p* < 0.0001) when compared to the three other groups, beginning as early as 2 days post-challenge (Fig. 4b). At 10 days post-challenge, bacterial burden was not statistically different between the Ir-*Cm* (+MDP) vaccinated group and the pre-exposed *Cm* group. A subsequent area under the curve (AUC) analysis of infectious burden similarly found no statistical difference in IFU between mice that were pre-exposed to *Cm* and vaccinated with Ir-*Cm* (+MDP), whereas AUC values were markedly higher in the three other groups (Fig. 4c).

**Fig. 5:**
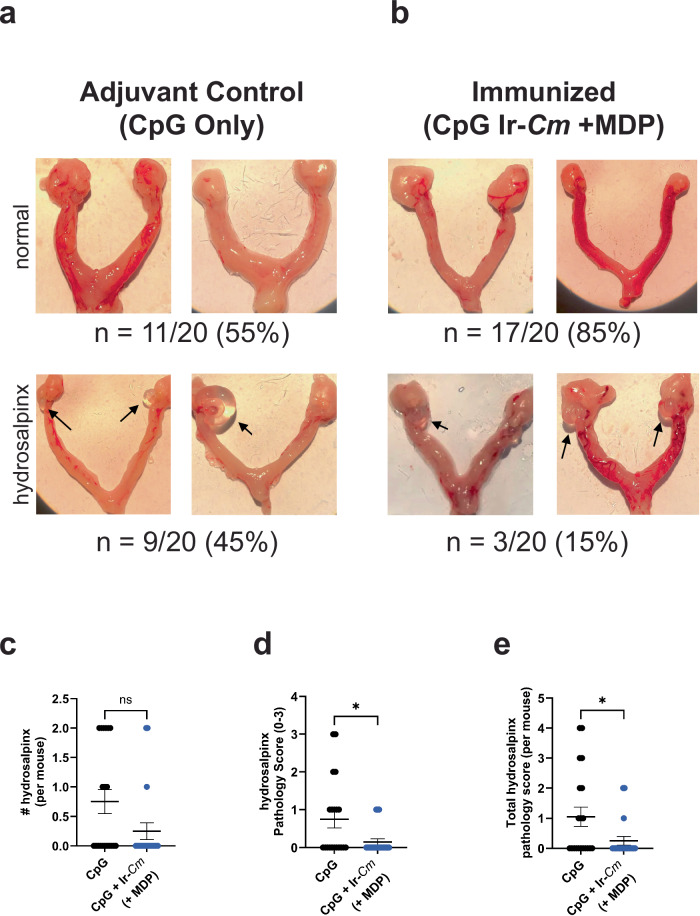
MDP+ whole-cell *Cm* vaccine reduces incidence and severity of hydrosalpinx. Representative images of normal (upper panels) or damaged (lower panels) upper genital tracts from adjuvant control (**a**) or immunized (Ir-*Cm* + MDP) (**b**) groups removed 30 days after intravaginal challenge with *Cm* (*n* = 20 mice/group). Examples of hydrosalpinx are indicated with black arrows. The percentage of animals with normal oviducts or hydrosalpinx from two combined experiments is presented below each figure. **c**–**e** Gross uterine pathology as determined by # of uterine horns displaying hydrosalpinx per mouse (**c**), highest pathology score for single uterine horns per mouse (**d**), and cumulative pathology score per mouse (**e**). Differences between immunized groups were assessed via the Mann–Whitney test. *p ≤ 0.05, ns not significant. Calculated *p* values for panels **c**–**e** are 0.065, 0.031, and 0.034, respectively.

To test the degree to which our irradiated whole-cell vaccine candidate influences the presentation of disease pathology post-challenge, we examined the oviducts from mice in our CpG/Ir-*Cm* (+MDP) vaccinated and our adjuvant control groups 30 days post-challenge. We found instances of hydrosalpinx, as defined as fluid-filled cysts, in roughly half of our adjuvant control group (45%, 9/20 mice) and 15% of our CpG/Ir-*Cm* (+MDP) vaccinated group (3/20 mice), which is significantly different at a level <0.1 (*p* value, 0.082; Mann–Whitney test) (Fig. 5a, b). If incidence was assessed per uterine horn (i.e., #hydrosalpinx per mouse), the two groups also differed by a significance value < 0.1 (Fig. 5c, *p* value 0.065; Mann–Whitney test). When assessed via pathology score, Ir-*Cm* (+MDP) immunized mice exhibited significantly lower scores than adjuvant controls (Fig. 5d, e; *p* values, 0.031 and 0.034, respectively; Mann–Whitney test). Altogether, these results demonstrate that Ir-*Cm* can confer a comparable level of protection with previous exposure to live *Cm*, that this protection is dependent on the presence of MDP during the irradiation process, and that vaccination appears to correlate with a reduction in disease severity.

**Fig. 6:**
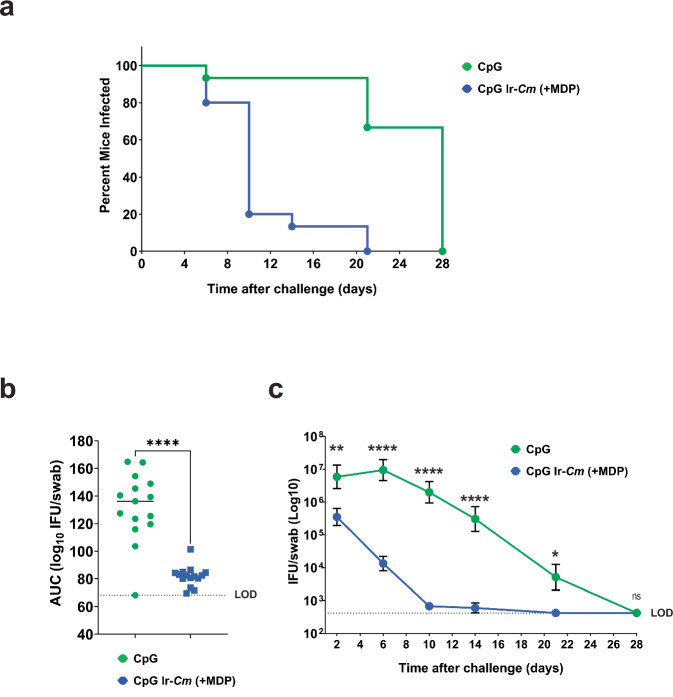
Protection conferred by Ir-*Cm* (+MDP) is long-lasting in mice. Four-month evaluation of Ir-*Cm* (+MDP) vaccine. Two groups of BALB/c mice were vaccinated with Ir-*Cm* (+MDP)/CpG or only adjuvant control (CpG alone control), (*n* = 15 mice per group) as described in Fig. 4, and challenged four months after receiving their last immunization. % infected over time (**a**), overall infectious burden calculated from an AUC analysis (**b**), and an average number (Log10) of IFU/mL of vaginal swab suspension over time (**c**) are shown. Groups were compared utilizing a Kruskal–Wallis test or two-way ANOVA with multiple comparisons, respectively. ****p < 0.0001, **p < 0.01, *p < 0.05, ns not significant, LOD limit of detection.

To assess the longevity of protection conferred by our vaccine candidate, we conducted an additional study using mice that were immunized with either CpG/Ir-*Cm* (+MDP) or adjuvant alone (CpG) and challenged with live *Cm* four months after the last vaccination dose was administered. Similar to our previous vaccination/challenge study, time to clearance was significantly reduced in mice immunized with CpG/Ir-*Cm* (+MDP) when compared to mice administered adjuvant alone (Fig. 6a). Bacterial burdens were also dramatically reduced in mice vaccinated with Ir-*Cm* (+MDP) (Fig. 6b) and significant differences (*p* < 0.005) in bacterial shedding were notable as early as two days post-challenge (Fig. 6c).

## Discussion

There is an urgent and ongoing need for a low-cost, effective, multivalent chlamydial vaccine. This has proved challenging, in no small part due to the fact that the evolutionary processes that led to *Chlamydia* spp. adapting to their intracellular niche predates the origin of most multicellular life on this planet^79^. This ancient group of bacteria evolved to the intracellular space long before the development of the innate and adaptive responses that vertebrates rely on to defend against invading pathogens. Despite this, some humans (and animals) are capable of mounting robust adaptive immunological responses to live and killed chlamydial pathogens. These responses can result in faster clearance^80^, but can sometimes come at the cost of exacerbating tissue damage and increasing disease-associated pathology^81^. Chronic disease resulting from *C. trachomatis* infection is associated with a failure of the adaptive immune response to the pathogen, bringing into question whether utilizing natural responses in humans to guide antigen selection for vaccine development is prudent^42^ (as discussed by Starnbach^68^).

A whole-cell vaccine has the benefit of providing numerous antigens for the development of immunological memory, provided they are not damaged during microbial inactivation. The physiological, immunological, and protection data we present here is consistent with the premise that combining the MDP complex with chlamydial EBs enhanced epitope protection during γ-irradiation without impairing the inactivation of the microbe. Because species in the genus *Chlamydia* lack a peptidoglycan sacculus^8,14,65^ the structural integrity of EBs relies almost entirely on a network of extensively crosslinked, cysteine-rich outer membrane proteins collectively referred to as the chlamydial outer membrane complex^82^. This unique physiological orientation also makes them particularly susceptible to ionizing radiation^83^. Importantly, MDP preserved the structural integrity of γ-inactivated EBs exposed to 20 kGy, and we therefore posit that MDP also serves to maintain the structure and integrity of EB polyvalent epitopes. Previous studies have also found that higher doses of γ-radiation can have damaging effects on lipopolysaccharide (LPS)^84–86^, which is a major component of Gram-negative bacterial outer membranes and a potent agonist of Toll-like receptor (TLR) 4^87^. While chlamydial lipooligosaccharide (LOS) has only minor TLR4-stimulatory potential^10^, chlamydial lipoproteins are highly stimulatory, and as such TLR2 plays an important role in the recognition of *Chlamydia spp*. in mammals^88^. We found that MDP did not enhance the TLR2 or TLR4 stimulatory potential of our inactivated EBs, and this is likely due to the fact that we were able to use a low enough dose of radiation to achieve complete sterilization without adversely impacting the lipid moieties of their LOS and lipoproteins. Based on these observations, we next tested the efficacy of a γ-irradiated, whole-cell *Cm* EB (+MDP) vaccine using CpG adjuvant to promote Th1-responses. Using a mucosal prime, systemic boost regimen, we found that the Ir-*Cm* (+MDP) vaccinations were well-tolerated, and resulted in significant, long-lasting protection against a subsequent challenge with live *Cm* four months after the final immunization. Prior studies have reported that EBs express antigens that can be deleterious to the induction of a protective immune response, however, we observed measurable protection in our inactivated EB vaccine and reduced disease pathology, indicating that any impact such antigens may have had were negligible in our study.

While there are inherent limitations with any model system, *Cm* was chosen for this work as it has previously been considered ideal for vaccine testing and antigen discovery, due to the characteristics of *Cm* murine infection closely matching the establishment/progression of infection and eventual disease pathology observed in humans infected with *C. trachomatis*^89^. *Cm* infection in female mice more closely mimics human *C. trachomatis* infections of the female genital tract: a single, low-dose vaginal inoculation results in ascension to the entire upper genital tract, resulting in enhanced bacterial burden, longer time-to-clearance, and enhanced pathology. By comparison, murine infections with *C. trachomatis* (i) can require multiple doses at higher inoculations, (ii) often utilize transcervical rather than vaginal challenge, (iii) are cleared much more rapidly than *Cm* infections, and (iv) exhibit limited pathology^89^. We also utilized progesterone in order to induce the murine vaginal epithelium to generate a thin layer of columnar epithelial cells instead of a mat of squamous cells laying on top of the columnar cells. This protocol increases the susceptibility of mice to chlamydial infection, but has been reported to skew subsequent immune responses towards a Th2-bias^90^.

The data we obtained from this vaccination/challenge study serves as proof of concept that MDP technology is an effective approach for the development of future whole-cell chlamydial vaccines. We were particularly encouraged to see that the Ir-*Cm* (+MDP) vaccine-induced both humoral and cell-mediated responses, similar to another successful whole-cell vaccination approach^41^. In our study, irradiated EBs induced elevated levels of *Cm*-specific serum and vaginal IgG responses regardless of whether MDP was used during the irradiation; however, IgG2a responses were greater (by ELISA) and vaginal IgA responses were greater (by western blot) in mice vaccinated with MDP-protected irradiated EBs. This is particularly important, as a successful *Chlamydia* vaccine must effectively recruit adaptive immune responses to the mucosal surfaces that are the primary site of infection^41,68^. The MDP-protected irradiated EB vaccine also induced higher levels of neutralizing Abs. These differences parallel the difference in efficacy induced by each vaccine, with the MDP-protected irradiated EB vaccine resulting in 78% clearance by day 10 post-challenge compared to 27% clearance in the mice given the Ir-*Cm* (−MDP) vaccine. While we did not directly identify the individual proteins recognized by the IgA present in our vaginal lavages, the ~40 kDa band is likely MOMP. We see significant differences in the recognition of this band between MDP +/− conditions as early as the first boost, and more pronounced after the second. More interestingly, only a single, ~60 kDa band appears to be prominent in the MDP- condition. There has been and continues to be much speculation concerning the potentially detrimental impact of Hsp60 (Heat-shock Protein 60) on the efficacy of any whole-cell chlamydia vaccine. Given our observations, it is tempting to speculate that the structural robustness of this protein enables it to endure the various inactivation methods employed by prior whole-cell vaccination approaches (and apparently our 1 kGy MDP- condition) better than chlamydial EB surface epitopes. Such an outcome could result in a ‘polyvalent whole cell vaccine’ driving a predominantly Hsp60-focused mucosal response, rather than the broader antigen-recognition response we observe in the MDP+ group in our vaginal lavage data. An evaluation of antigens uniquely reactive to vaginal IgA from mice immunized with Ir-*Cm* (+MDP) is currently ongoing.

While there is some disagreement in the field as to whether high antibody titers of *Chlamydia*-specific antibodies contribute to a productive response to infection^91^, it is widely accepted that CD4+ T cells often play a role in long-term protection. In addition to humoral responses, the cytokine profiles we obtained from our splenocyte restimulation study matched with what is known about protective responses to chlamydia infections. Splenocytes from animals vaccinated with Ir-*Cm* (+MDP) showed enhanced levels of IFNγ, TNFα, IL-18, IL-12, and IL-2; a cytokine profile consistent with clearance via a robust cell-mediated, Th1-focused response. The marked increase in IL-18 is striking. IL-18 has been shown previously to induce IFN-γ production in B, T, and NK cells^92^ but has also been demonstrated to have no effect on clearance of *Cm* in a murine lung infection model^93^. By contrast, splenocytes harvested from animals previously exposed to live *Cm* exhibited high levels of IFNγ and TNFα, but also IL-6, IL-10, IL-17A, IL-1β, and IL-4; a cytokine profile consistent with a more balanced Th1/Th2/Th17 response. Chlamydia-infected cervical cells are known to secrete IL-1, IL-6, IL-8, and IL-10^94^, and cytotoxic CD8+ T cells and other immune cells readily infiltrate tubal tissue implants infected with *C. trachomatis*, with infiltrated cells expressing IFNγ, IL-2, and IL-6. IL-6-mediated signaling pathways have been reported to limit *Cm* infection while also exacerbating pathology in mice^95^, and *Ct*-induced IL-6 signaling is significantly reduced when *Ct* is inactivated via UVC^96^. Moreover, studies confirmed the presence of CD8+ T cells in the fallopian tubes and their association with pathology in primates and guinea pigs following infection with *C. trachomatis*^97,98^. IL-10 acts directly to inhibit IL-12, inhibits the ability of antigen-presenting cells to present antigens to T cells^99^, and high levels of IL-10 detected in the cervical lavages of women with *C. trachomatis* infections have been linked to infertility^100^.

With the caveat that our splenocyte assay data was only conducted once (five mice per group), the data raises an interesting query. While preservation of protein structure by MDP would explain the enhancement that we observe in *Cm*-specific IgG and IgA in serum and vaginal washes, it is less clear why protecting surface antigens from ROS-induced damage would enhance T cell responses. Prior research examining differences in cell-mediated responses to killed vs live chlamydial EBs offers potential explanations for this puzzling finding, with the qualification that our work is the first to examine these phenotypes in EBs killed via ionizing radiation. In vivo, living and killed EBs have been demonstrated to differ in their ability to induce DC and neutrophil infiltration^101^ and target different T cell populations^41^. Prior studies utilizing in vitro stimulation assays have also demonstrated that DCs pulsed with live vs UVC- and/or heat-killed EBs exhibit differences in DC maturation, expression of MHC class II and various cytokines, and efficiency of antigen presentation^51,56,101^. Interestingly, one study found that a major difference in antigen presentation via MHC class II in DCs pulsed with live or killed EBs was an absence of any chlamydial surface antigens presented by DCs exposed to EBs inactivated by heat/UVC^56^. In light of these prior observations, we favor the hypothesis that the MDP-antigen preservation effect could also influence how efficiently DCs ingest, process, and present antigenic peptides. Alternatively, given that some cytokines in our splenocyte assay gave different responses if they were re-stimulated with Ir-*Cm* +/− MDP, there is the potential that the MDP peptide could be eliciting some responses to itself and we are observing a minor recall response from *MDP-*specific CD4 memory T cells. The potential also exists that MDP is behaving as a mild adjuvant for certain cytokines, via unknown effects on antigen-presenting cells.

Previous studies have found that rather than promote protection, pre-exposure to formalin-fixed or irradiated chlamydial EBs actually exacerbates subsequent infection^41,44,46,53–55^, due to irradiated EBs being incorrectly targeted to tolerogenic dendritic cells, rather than to immunogenic uterine dendritic cells^41^. Given these observations, we suspect that the breakdown of EB outer membrane components during irradiation may influence their ability to successfully traffic to immunogenic dendritic cells, or that radiation significantly alters their structurally important epitopes. Experiments more stringently investigating the mechanism(s) of protection of the Ir-*Cm* (+MDP) vaccine are currently underway. A number of irradiated, whole-cell chlamydia vaccine candidates in the past have been generated using sterilization with ultraviolet C (UVC) light^7^. While UVC-inactivation is relatively inexpensive and widely utilized, it has several disadvantages, the most pressing being that UVC does not uniformly penetrate biological samples^102^, which inevitably leads to uneven UVC dosing in a given preparation. This deficiency is compounded by the fact that there is currently no standardized UVC inactivation method utilized across the field, potentially offering an additional explanation for the variability in experimental outcomes between research groups^7^. By comparison, γ radiation exhibits high penetrance in biological samples in aqueous environments^103^, ensuring that all bacteria within a sample have been uniformly exposed. Whereas γ-rays are a form of ionizing radiation and UVC is non-ionizing, both forms of radiation generate ROS, which specifically damage proteins. Manganese (Mn) antioxidants catalytically scavenge superoxide generated by γ-rays^103,104^ and singlet oxygen generated by UVC^105,106^. Irradiated chlamydial EBs have long been used as controls for a number of immunization studies, or as tools for assessing immunological responses, such as whole-cell ELISAs. Given our observations, it may be prudent to re-evaluate their usage until the impact of free oxygen radicals generated during the inactivation procedure on EB surface epitopes has been directly investigated.

Previous research has explored γ-irradiation for studying the immunostimulatory potential of inactivated chlamydial EBs, yielding varied outcomes. For instance, Colley et al. found that ocular exposure to live (but not irradiated) *C. trachomatis* sensitizes mice to re-exposure to either live or irradiated *Chlamydia*^71^. MacDonald et al. demonstrated that after vaccination with γ-irradiated EBs, *C. trachomatis*-specific antibodies increased in vaccinated animals but animals had a longer course of disease and more ocular discharge than naive animals^44^. Nelson et al. utilized γ-radiation to demonstrate that irradiated *Cm* was capable of restoring *C. trachomatis* infectivity in IFN-γ treated cells^107^. A confounding factor in these studies is the wide range of radiation doses used: 1000 Gy, 10,000 Gy, and 3280 Gy, respectively. Furthermore, the conditions under which irradiation was conducted varied significantly from our approach. Generally, a higher dose of γ-radiation correlates with increased oxidative damage to cellular macromolecules. However, the extent of protein and DNA damage from a specific radiation dose can vary based on the state of the sample—aqueous, desiccated, or frozen. Notably, Nelson and MacDonald irradiated EB cells in a frozen state, with MacDonald further desiccating the EBs post-irradiation; desiccation would exacerbate oxidative damage to irradiated cell surfaces. This contrasts with our methodology, where we employed aqueous MDP-irradiation conditions, which, as our results indicate, have a different impact on the preservation of antigenic structures and overall vaccine efficacy.

We are currently optimizing our MDP-irradiation method for vaccine preparation for the human pathogen, *C. trachomatis*. The multivalent nature of the vaccine design would theoretically broaden the number of *C. trachomatis* serovars/strains covered by a single vaccination strain, while its inactivated status effectively removes the majority of the immunosuppressive features of the pathogen that require protein synthesis and an active type 3 secretion system (T3SS). Enhanced coverage could alternatively be achieved by generating inactivated strain cocktails utilizing several different *Ct* serovars, or simply harnessing the advances that have been made in chlamydial genetics over the last few years^108^ and generating hybrid vaccine strains expressing antigens (such as MOMP) normally unique to individual serovars. While additional studies will likely be required to establish effective dosing, and adjuvant optimization, given the method for generating our vaccine is relatively straightforward, it can be adapted to a range of additional *Chlamydia* species. Chlamydial EBs are prepackaged with a number of proteinaceous effector proteins that are delivered to host cells rapidly via the T3SS upon the microbe contacting the cell surface. Given that this process presumably does not require transcription or translation and that MDP protects many proteinaceous components of the chlamydial cell during γ-irradiation, it is unclear whether the delivery of these early effectors may be unaffected. The degree to which these ‘inactivated’ pathogens are able to initially enter host epithelial and immune cells and carry out some rudimentary functions is currently being investigated.

In summary, in this study, we explored the use of the Manganese (Mn^2+^) Decapeptide (DEHGTAVMLK) Phosphate (MDP) complex in developing a novel whole-cell *Chlamydia* vaccine. This approach capitalizes on previous research on MDP’s radiochemistry and structure^83,109^ and the ability of MDP to protect antigenic structures from ROS-mediated radiation damage^69,110,111^. Our results underscore three notable findings: First, we demonstrated that our method of gamma irradiation in the presence of MDP not only preserved the structural integrity of irradiated EBs but also protected the epitopes in *Cm* without preventing genome inactivation. This ensures the vaccine’s antigenic potency, which is crucial for eliciting a robust immune response in radiation-inactivated vaccines. Second, our immunization data showed that mice vaccinated with *Cm* irradiated in the presence of MDP achieved high levels of *Cm*-specific antibodies to a broad array of antigens and generated a beneficial cell-mediated response upon re-stimulation. Moreover, our challenge data indicated that vaccination with Ir-*Cm* (+MDP) not only increases the number of recognized antigens but also that the recognition of many of these antigens is likely protective, as the vaccine confers a level of protection similar to pre-exposure to live *Cm*. Third, immunization studies with Ir-*Ct* (+/−MDP) will likely be useful for identifying protective target antigens by comparing differences in the recognized proteins between the two vaccination groups. Our findings thus pave the way for a new generation of whole-cell, multivalent *Chlamydia* vaccines, offering a promising strategy to combat a major global health challenge.

## Methods

### Reagents

The synthetic peptide DP1 decapeptide H–Asp-Glu-His-Gly-Thr-Ala-Val-Met-Leu-Lys–OH^69^ was custom-synthesized and refined to 95% purity by Sigma-Aldrich (The Woodlands, TX, USA) and concentrations of stock DP1 solutions were confirmed by LC-MS. Manganese Chloride, Potassium Phosphate dibasic trihydrate (K_2_HPO_4_·3H_2_O), and Potassium Phosphate monobasic, (KH_2_PO_4_) were all purchased from Sigma. CpG ODN 1018 (InvivoGen) was used for all immunization studies.

### Cell culture and growth conditions

HeLa and L2 mouse fibroblast cells were provided by Anthony Maurelli (University of Florida). Cells were cultured in T-175 flasks (BD Falcon) using Dulbecco’s Modified Eagle Medium + GlutaMAX (Gibco) (DMEM) supplemented with 10% heat-inactivated Fetal Bovine Serum (FBS, HyClone) at 37 °C with 5% CO_2._ Cells were checked for mycoplasma contamination after 2 passages from the initial liquid nitrogen thaw, and every subsequent 10 passages.

### Cell infection and bacterial growth conditions

*C. trachomatis* serovar L2 strain 434/Bu was provided by Harlan Caldwell (Rocky Mountain Laboratories, MT, USA). *Cm* strain Nigg^112^ was provided by Toni Darville (University of North Carolina, NC, USA). Whole-cell lysate (‘crude’) freezer stocks of chlamydial EBs were generated from HeLa cells 40 h post-infection stored at −80 °C in sucrose phosphate glutamic acid buffer (7.5% *w*/*v* sucrose, 17 mM Na_2_HPO_4_, 3 mM NaH_2_PO_4_, 5 mM L-glutamic acid, pH 7.4) until use. Stocks were titered via inclusion forming unit (IFU) assay before use. For generating EB preparations for irradiation and vaccination experiments, 8 T-175 flasks (BD Falcon) were infected with chlamydial crude stocks at a MOI of ~1. Flasks were placed on a rocking platform for 2 h at 37 °C with 5% CO_2_, after which time the initial inoculate was removed and replaced with DMEM supplemented with 10% heat-inactivated FBS at 37 °C with 5% CO_2_. At 40 hpi, cell monolayers were detached by glass beads, lysed by sonication, and centrifuged at 10,000 rpm on a tabletop centrifuge for 60 min at 4 °C. Pellets were resuspended in 30% Percoll/70% HEPES by volume before spinning at 15,400 rpm (30,000 g) for 30 min at 4 °C. The top layer of the resulting gradient was removed, before diluting and resuspending the bottom band in the same volume of HEPES. The suspension was then spun at 15,400 rpm for another 30 min at 4 °C. The pellet was washed with HBSS before a final spin at 15,400 rpm for 30 min at 4 °C. The pellet was then resuspended in sterile endotoxin-free PBS for the addition of subsequent MDP components prior to irradiation.

### Chlamydial IFU assays

Ninety-six well tissue culture-treated plates (Fisher) were seeded 24 h prior to IFU assays with 200 µL per well of a 200,000 L2 cells/mL suspension in DMEM/10% FBS (~40,000 cells per well). On the day of the assay, bacterial suspicions were thawed on ice and then serially diluted in the infection medium [DMEM, 10% FBS, MEM Non-Essential Amino Acids (Sigma), 0.5 µg/mL cycloheximide]. Spent media was removed from the 96-well plate and 200 µL of each chlamydial dilution was added to each well, with each dilution conducted in duplicate. Plates were then spun in a tabletop centrifuge (Eppendorf) at 3000 rpm at 35 °C for 1 h to synchronize infection and ensure that all infectious EBs come into contact with cell monolayers. Plates were then incubated for 24–28 h at 37 °C 5% CO_2_. At the desired hpi, the infection medium was aspirated, infected cells were fixed/permeabilized by the addition of 200 µL of ice-cold methanol and incubated at room temperature for ~10 min. The methanol was then aspirated and one drop of Pathfinder *Chlamydia* Culture Confirmation System (BioRad) was added to each well. Plates were incubated at room temperature in the dark for 30 min, after which time the antibody staining solution was removed, wells were gently washed three times with deionized water, and one drop of glycerol mounting medium was added to each well. Plates were inverted, stored at 4 °C, and examined under an epifluorescence microscope (Olympus) within 24 h. Total IFU was calculated by counting the number of visible inclusions for 40 different imaging fields using the 40× objective.

For the assessment of bacterial burden during immunization/challenge studies, if no inclusions were observed in 40 separate imaging fields at the lowest sample dilution, the rest of the coverslip was scanned. As we never observed additional inclusions in samples that came back negative after the first 40 fields of view, we decided to be conservative and report these values at the assay’s originally calculated LOD (~400 IFU). For the assessment of inactivation of irradiated organisms, bacteria were centrifuged onto HeLa cell monolayers plated on 24 well coverslips, and allowed to incubate for 24 h. Cells were then fixed and stained utilizing either the Pathfinder Detection Kit (Bio-Rad) or a MOMP monoclonal antibody (followed by an Alexa Fluor-conjugated secondary). Coverslips were imaged utilizing a Zeiss 700 confocal microscope and examined for the presence of inclusions containing multiple organisms. For each 12 mm coverslip, all fields of view (first pass ~1177 via eyepiece, second pass ~10,970 at 1× zoom in confocal mode) were assessed for the presence of replicating organisms.

### *Chlamydia* irradiation procedure

For irradiation experiments, ~10^7^–10^9^ *Cm* or *C. trachomatis* Percoll gradient-enriched EBs were subjected to γ-irradiation on wet ice (0 °C) in 0.5 mL o-ring polypropylene tubes using a ^60^Co source (dose rate disclosure is not allowed). Bacteria were irradiated in the presence or absence of MDP: 3 mM DP1 [DEHGTAVMLK], 25 mM potassium phosphate, pH 7.4, and 1 mM MnCl_2_, with MnCl_2_ added last, as previously described in ref. ^111^. Prior to and after end-point irradiation for all experiments conducted and vaccinations prepared, aliquots of the EB suspension were collected to determine viable bacterial numbers (pre- and post-irradiation) via the IFU assay. For all *Cm* vaccination studies, a small 10 μL aliquot was taken from the suspension before irradiation to determine the IFU/mL, and another 100 μL was taken after irradiation (1 kGy) for serial dilution and IFU assay to confirm EB inactivation via IFU assay and subsequent assessment via confocal microscopy.

### Microscopy and imaging studies

For the preparation of samples for imaging via transmission electron microscopy, live and irradiated *C. trachomatis* EBs were fixed in 2% formaldehyde and 2% glutaraldehyde in cacodylate buffer, (CB, 0.2 M, pH 7.2) overnight at 4 °C. Bacterial cells were pelleted by centrifugation, and embedded in 2% low-temperature gelling agarose to aid in subsequent processing. Samples were then post-fixed in 2% OsO_4_ in CB for 1 h at room temperature followed by 3 × 10-min washes in CB. Each sample was then dehydrated in a graduated series of ethanol, infiltrated with Spurr’s epoxy resin (Electron Microscopy Sciences, Hatfield, PA), and polymerized at 70 °C for 12 h. Following polymerization, ultrathin sections (70–80 nm) were cut on a Leica UC6 ultramicrotome (Leica Microsystems, Inc, Buffalo Grove, IL) and collected on 200-mesh copper grids. Grids were post-stained in 2% aqueous uranyl acetate for 15 min and Reynold’s lead citrate for 5 min. Grids were then examined on a JEOL JEM-1011 transmission electron microscope (JEOL USA, Inc., Peabody, MA), and images were captured on an Advanced Microscopy Techniques 4MP digital camera (AMT Corp., Woburn, MA).

### TLR reporter assays

HEK-Blue cells expressing either the human TLR2 or TLR4 receptor and carrying the NF-κB SEAP (secreted embryonic alkaline phosphatase) reporter gene (InvivoGen) were used according to the manufacturer’s instructions and adapted to assess TLR-specific NF-κB activity induced by live or irradiated *Cm*. Briefly, 3 × 10^5^ cells/mL of HEK-Blue-hTLR2, HEK-Blue-hTLR4, or -Null1 cells were plated in 96-well plates (total reaction volume of 200 µL per well [∼6.0 × 10^4^ cells per well]) and allowed to settle/adhere overnight at 37 °C. Media was then removed and replaced with 200 µL of medium containing *Cm* at MOIs of ~2, 0.2, or 0.02. Plates were then centrifuged for 1 h at 2000×*g* and subsequently incubated in a CO_2_ incubator at 37 °C. Cell supernatants were collected at 24 hpi and 48 hpi for subsequent analysis of SEAP activity. A colorimetric reporter assay was then utilized to quantify the abundance of SEAP in cell supernatants. Twenty microliters of supernatant collected from infected cells were added to 180 µL of the SEAP detection solution (InvivoGen), followed by incubation at 37 °C for ∼6 h. SEAP enzymatic activity was then quantified using a plate reader set to 650 nm. Infected cells were compared to uninfected (media) controls and cells treated with the known TLR2 and TLR4 signaling ligands (Pam3CSK4 and LPS, respectively) were used as positive controls. To ensure that changes in alkaline phosphatase activity were TLR-dependent under each of the experimental conditions tested, all experiments were carried out in parallel in the HEK-Blue-Null1 cell line, which contains the empty expression vector but lacks hTLR2/4. HEK-Blue NOD1 SEAP reporter assays were carried out in three separate experiments, statistical analysis was conducted by two-way ANOVA, and significance values were analyzed by utilizing Sidak’s multiple-comparison test. Values plotted are means of the raw optical density at 650 nm (OD_650_) measurements.

### Murine immunization and challenge studies

Four-week-old BALB/C Mice (10 per group) were vaccinated i.n. on d0 with either PBS, adjuvant (Class C CpG), or adjuvant with ~10^7^ *Cm* EBs irradiated in the presence or absence of MDP (Supplementary Fig. 6). Subsequent immunizations (~10^7^ *Cm*) were administered s.c. at two (d14) and four (d28) weeks after the initial vaccination. As a positive control, an additional group of 10 mice was inoculated with 10^5^ IFU of live *Cm* administered on d28 by the i.u. route and allowed to naturally clear the infection. Blood and vaginal lavages were collected on d24, d38, and d56 from the lateral saphenous vein and by flushing the vagina in and out 5 times with 30 µL of sterile PBS and combining 3 separate washes from each time point (~100 µL final sample). For mice designated for splenocyte analysis, animals were vaccinated as described above, but mice in the positive control group were administered live *Cm* by i.u. at day 10. All mice were then sacrificed at d38 (anesthetized with ketamine/xylazine i.p. followed by cervical dislocation), and the spleens were removed for processing.

Three weeks after the final immunization, all mice were given a single dose of 2.5 mg of medroxyprogesterone (Depo-Provera^®^; Upjohn, Kalamazoo, MI) s.c., as previously described in ref. ^113^. Seven days later, mice from all groups were vaginally challenged with 10^5^ live *Cm*. Vaginal swabs were collected every 4 days for quantitative *Cm* culture. Thirty days post-challenge, mice from the Ir-Cm (+MDP) and CpG alone groups were anesthetized with ketamine/xylazine (i.p.), and sacrificed by cervical dislocation. While pathology is often assessed as late as 60 days post-infection with *Cm*^114,115^, the shutdown of facilities following the onset of the COVID-19 pandemic required us to limit our duration to 30 days, and limit our pathology comparisons between only adjuvant control and Ir-*Cm* (+MDP) immunized animals. Upper reproductive tracts were harvested for pathology, and evaluation of incidence and severity of hydrosalpinx, which was assessed using a four-point scale (0: normal; 1: hydrosalpinx clearly visible but smaller than that of the ovary; 2: hydrosalpinx similar in size to the ovary; 3: hydrosalpinx larger than the ovary. The experiment was repeated to test reproducibility and increase the statistical power. A similar challenge study was conducted to test the duration of vaccine-mediated protection in which Ir-*Cm* (+MDP) with CpG or CpG alone was administered to two sets of mice as above, and the mice were challenged with *Cm* at 4 weeks (*n* = 10 mice/group) or 4 months (*n* = 15 mice/group) after the final immunization. A higher number of mice was used in the second group to account for reduced susceptibility to *Cm* in older mice. This work was covered under the USU Institutional Animal Care and Use Committee (IACUC) approved protocol MIC22-977 entitled “Development of vaccines against sexually transmitted bacterial urogenital infections (mouse)”. We have complied with all relevant ethical regulations concerning the animal studies described in this manuscript.

### Antibody analysis and neutralization assay

Serum and mucosal antibodies collected from vaccinated and control mice on day 38 were tested for specificity against *Cm* whole-cell lysate antigen (*Cm* EBs heated to 95 °C for 5 min) via western blot. Antigen load was controlled through monitoring of protein concentrations via nano-drop (242 µg/mL) and loaded onto 4–20% tris-glycine ZOOM SDS-Page gels via a single, wide well that was subsequently run prior to transfer to nitrocellulose. Nitrocellulose filters were blocked overnight in PBS-tween-20 nonfat-dry milk (0.1% and 5%, respectively). Filters were incubated with serum or vaginal lavages from individual mice diluted in PBST utilizing a Mini-PROTEAN® II Multiscreen Apparatus (Bio-Rad) followed by incubation with goat anti-mouse IgG-HRP (Bio-Rad 172–1011) or goat anti-mouse IgA-HRP (Southern Biotech 1040-05) diluted in PBST. Uncropped and unprocessed blots used to construct Fig. 2d are presented in Supplementary Fig. 9. Quantification of western blot protein band intensities was carried out utilizing ImageJ, as previously described in ref. ^116^. Comparisons between +MDP and −MDP samples are expressed as a ‘ratio of arbitrary units’, which represents the ratio of ‘net band’ to ‘net loading control’. These values were derived by first calculating an intensity value (‘net band’) for each band of interest by deducting individual lane backgrounds from raw band intensity measurements. A net loading control was similarly calculated, and then the ratio of the net band to net loading control was calculated for each protein of interest.

An enzyme-linked immunosorbent assay (ELISA) was used to measure *Cm*-specific serum antibody titers as follows: 0.1 mL of 10 µg/mL whole *Cm* EBs (1 µg/well) were added to microtiter plates (Immulon 2HB) and incubated overnight at 4 °C (one plate per antibody isotype). Unbound EBs were removed by inverting each plate and tapping on a paper towel followed by the addition of 0.2 mL block (PBS-tween 20-FBS, 0.05% and 3%, respectively) to each well, and plates were incubated overnight at 4 °C. The next morning block was removed from the ELISA plates and washed 4X with PBST, and 2-fold dilutions of d38 sera collected from individual mice diluted in PBST were added to each well (volume, 125 µL) starting with a 1:1000 dilution. Plates were then covered and incubated at RT for 1 h. Plates were washed 4× in PBST and 100 µL of secondary antibody diluted in PBST was added to each appropriate well and incubated RT for 1 h. Secondary antibodies were prepared in 10 mL PBST: Goat Anti-Mouse IgG, Human ads-HRP: 1:10,000, Goat Anti-Mouse IgG_1_, Human Ads-HRP: 1:10,000, Goat Anti-Mouse IgG_2a_, Human Ads-HRP: 1:5000. Plates were washed 4× in PBST and incubated with TMB substrate solution prepared according to the manufacturer’s instruction (Bio-Rad). Reactions were stopped by the addition of 0.1 mL of 1 N H_2_SO_4_ to each well and plates were read at an absorbance of 450. Titers were determined by taking the average absorbance values from antigen-only control wells + 3 standard deviations (3 SD). Absorbance values at or above this value were deemed the ‘titer’. Pooled serum from animals immunized with CpG-alone was used to establish the assay’s baseline, which was found to have an LOD of 1:1000; log 3.

For serum neutralization assays, pooled serum was serially diluted in PBS (1:10–1:10,000) to total volumes of 500 µL. Approximately 10^7^ live *Cm* EBs suspended in 500 µL of PBS were added to each dilution, resulting in a 1 mL total EB/serum mix. The serum/EB mixture was incubated at 37 °C with rocking for 30 min. Two hundred microliters (~10^6^ EBs) were then added to each well plate and incubated at 37 °C with rocking for 2 h. Serum/EB suspensions were then aspirated and replaced with 1 mL of fresh infection media, and plated cells were then incubated for 24 h at 37 °C. The next day, the infection medium was aspirated, cells were fixed with methanol, and inclusion stains for calculating IFU counts.

### Splenocyte assay

For splenocyte analysis, female BALB/c mice were vaccinated as described above, but mice in the positive control group were administered live *Cm* by i.u. at day 10 (*n* = 5 for all groups except CpG IR-*Cm*; *n* = 4). All mice were then sacrificed at d38 (anesthetized with ketamine/xylazine i.p. followed by cervical dislocation), and the spleens were removed for processing. Cell suspensions were generated in RPMI 1640 utilizing a 40 µm cell strainer and sterile plunger. Red blood cells were lysed utilizing ACK lysis buffer, remaining cells were pelleted via centrifugation at 500×*g* for 5 min. Remaining cells were resuspended in RPMI 1640 supplemented with 10% FBS and 1% penicillin/streptomycin (Gibco). Resuspended cells were plated at ~10^6^ cells/mL per well in a 24 well plate and either left unstimulated or stimulated with ~5 × 10^5^ *Cm* EBs that had been irradiated in the presence or absence of MDP. After 3 days of incubation under these conditions (at 37 °C, 5% CO_2_), plates were spun at 500×*g* for 5 min to pellet cells in suspension, and cell supernatants were then collected and then stored at −80 °C. Subsequently, cytokines in the supernatants were assayed using a 17-plex bead array (ThermoFisher) performed according to the manufacturer’s instructions. The assay was read on a FlexMAP 3D instrument (Luminex) and data was analyzed using Bio-Plex Manager v6.2 (Bio-Rad). Results below the lower limit of quantification (LLOQ) were imputed as half the LLOQ.

## Supplementary information

Supplementary Information

## Data Availability

All data generated in this manuscript will be made available without restriction upon request. This includes all PRISM files, all reporter cell line SEAP data, and chlamydial IFU calculations.
