# Supplementary material for: Irradiated whole cell Chlamydia vaccine confers significant protection in a murine genital tract challenge model
Source: NPJ Vaccines. 2024 Nov 11;9:207. doi: 10.1038/s41541-024-00968-z (PMC11554809; doi:10.1038/s41541-024-00968-z)
Supplement: Supplementary file 1 — Supplementary Information [file 41541_2024_968_MOESM1_ESM.pdf]

SUPPLEMENTARY DATA FIGURES / TABLES

FIGURE LEGENDS

Supplementary Figure 1. γ-irradiation dose-response kill curves for *C. trachomatis*. (a) High titer stocks of *C. trachomatis* (serovar L2 strain 434/Bu) EBs were prepared via Percoll gradient enrichment and subjected to increasing doses of γ–radiation in the presence or absence of MDP. **(b)** A direct comparison of survival between *C. trachomatis* (serovar L2 strain 434/Bu) and *C. muridarum* (strain Nigg.) EBs under increasing doses of γ–radiation in the presence or absence of MDP. Each data point represents the average of two technical replicates. All survival curves displayed are from separate experiments. LOD; limit of detection.

Supplementary Figure 2. Live and γ-irradiated *Cm* EBs exhibit differential signaling through Toll-like receptor 2. HEK293 hTLR2 (**a,b**) and hTLR4 (**c,d**) reporter cells were infected with live or irradiated *C. muridarum* in the presence or absence of MDP at MOIs of 2, 0.2, and 0.02. Supernatants were collected at 24 (**a, c**) and 48 (**b, d**) hours post-infection (hpi) and tested for SEAP activity via a colorimetric assay. Data presented are the mean of three biological replicates and error bars represent standard error of the mean. Significance was assessed by two-way ANOVA with multiple comparisons. **, $p< 0.01$; ns, not significant.

Supplementary Figure 3. Intranasal prime subcutaneous boost (x1) immunizations with whole-cell *C. muridarum* vaccine irradiated in the presence of MDP (Ir-*Cm* +MDP) enhances the production of EB-specific serum IgG and IgA. Western blots against fractionated *C. muridarum* EB lysates showing *Cm*-specific serum IgG **(a)** and vaginal IgA **(b)** from individual mice vaccinated with *C. muridarum* irradiated (Ir-*Cm*) in the presence or absence of MDP, adjuvant (CpG) alone, or PBS (naïve mice). Serum and vaginal washes were collected

on d24; 10 days after the second immunization and tested via WB at a 1:10,000 and 1:200 dilution, respectively. Dotted lines are used to delineate between experimental groups on WBs. **(c)** Quantitative analysis of band intensity for the six most prominent bands from western blots using vaginal IgA (as indicated by asterisks in **b**). The mid-line indicates the mean of all data points and error bars represent standard error of the mean. Significance was assessed via Mann-Whitney test. ****; p< 0.0001, ***; p< 0.001, **; p < 0.01, ns; not significant.

Supplementary Figure 4. Intranasal prime subcutaneous boost (x2) immunizations with whole-cell *C. muridarum* vaccine irradiated in the presence of MDP (Ir-*Cm* +MDP) enhances the production of EB-specific serum IgG and IgA. Western blots against fractionated *C. muridarum* EB lysates showing *Cm*-specific serum IgG **(a)** and IgA **(b)**, and vaginal IgG **(c)** from individual mice vaccinated with *C. muridarum* irradiated (Ir-*Cm*) in the presence or absence of MDP, adjuvant (CpG) alone, or PBS (naïve mice). Serum and vaginal washes were collected on d38; 10 days after the final immunization and tested via WB at a 1:10,000 and 1:200 dilution, respectively. Dotted lines are used to delineate between experimental groups on WBs.

Supplementary Figure 5. Principle component analysis (PCA) of splenocyte cytokine production demonstrates unique clustering of Ir-*Cm* (+MDP) compared to other vaccination and pre-exposure groups. A PCA was conducted utilizing the levels of 11 cytokines (presented in **Figure 3** and **Supplementary Figure 5**) measured when splenocytes were re-stimulated with *Cm* EBs that were irradiated in the presence or absence of MDP. Panel **a** displays each of the five replicates per vaccination / pre-exposure group and panel **b** displays the individual cytokine vectors reflected in the grouping data.

Supplementary Figure 6. Few differences in cytokine secretion were observed between splenocytes stimulated with Ir-*Cm* (-MDP) and Ir-*Cm* (+MDP) EBs. **a)** Spider Plots demonstrating a side-by-side comparison of all cytokines examined between splenoctyes stimulated with *Cm* irradiated either in the presence / absence of MDP. The plots displayed in the first row ("Ir-Cm (+MDP) Stimulated") are the same ones presented in **Fig. 3b**. **b)** Levels of IL-2 and IL-1B produced from splenocytes restimulated with Ir-*Cm* (+/- MDP). Error bars represent standard error of the mean. Groups were compared utilizing 2way ANOVA with multiple comparisons. ****; $p < 0.0001$, ***; $p < 0.001$, **; $p < 0.01$, *; $p < 0.05$.

Supplementary Figure 7. Vaccination / Challenge Study Design.

Supplementary Figure 8. Ir-*Cm* (+MDP) enhances bacterial clearance from the genital tract in a murine vaccination / challenge model. % infected **(a)**, average IFU/mL vaginal swab suspension over time **(b)**, and overall infectious burden calculated from an Area Under the Curve (AUC) analysis **(c)** from each separate experiment used in **Figure 4** are shown. Each group consisted of 10 BALB/C mice, and differences among the groups were compared as described in **Figure 4**. Statistical significance readouts are shown for comparisons to the group of mice that was pre-exposed to *C. muridarum* (positive control). ***; $p < 0.001$, ns; not significant. LOD; limit of detection.

Supplementary Figure 9. Uncropped and unprocessed scans of western blots used in Figure 2d. Western blots against fractionated *C. muridarum* EB lysates showing vaginal *Cm*-specific IgA from individual mice vaccinated with *C. muridarum* irradiated (Ir-*Cm*) in the presence or absence of MDP **(a)**, adjuvant (CpG) alone, or PBS (naïve mice) **(b)**. Vaginal washes were collected on d38; 10 days after the final immunization and tested via WB at a

1:200 dilution. Blots are shown as imaged on an Amersham Imager 680 in combined mode (left columns) and chemiluminescence only mode (right columns).

Supplementary Figure 10. Graphical Abstract. An efficacious, irradiated, whole-cell Chlamydia vaccine is made possible by the utilization of Manganese Decapeptide Phosphate (MDP) antioxidant complex. When bacteria are exposed to ionizing radiation in aqueous environments, hydroxyl and peroxyl radicals are produced. Hydroxyl radicals directly damage genetic material whereas peroxyl radicals predominantly damage proteins and lipids. High doses of ionizing radiation can lead to lipid peroxidation and the molecular breakdown of biological membranes. Chlamydia EBs are particularly susceptible to ionizing radiation, as they lack the structural rigidity generally conferred by a peptidoglycan layer. When microbes are irradiated in the presence of MDP antioxidant complex, their surface lipids and proteins are protected from damage induced by peroxyl radicals while damage to genomic DNA by hydroxyl radicals continues unabated. Given the high penetrance of γ-radiation, this technique allows for the sterilization of densely-packed, bacterial samples with relatively low radiation doses, while simultaneously preventing damage to surface epitopes important for immune recognition and the development of immunological memory.

Supplementary Table 1. Mantel-Cox tests for all groups presented in the % infected study (Fig. 4a).

a

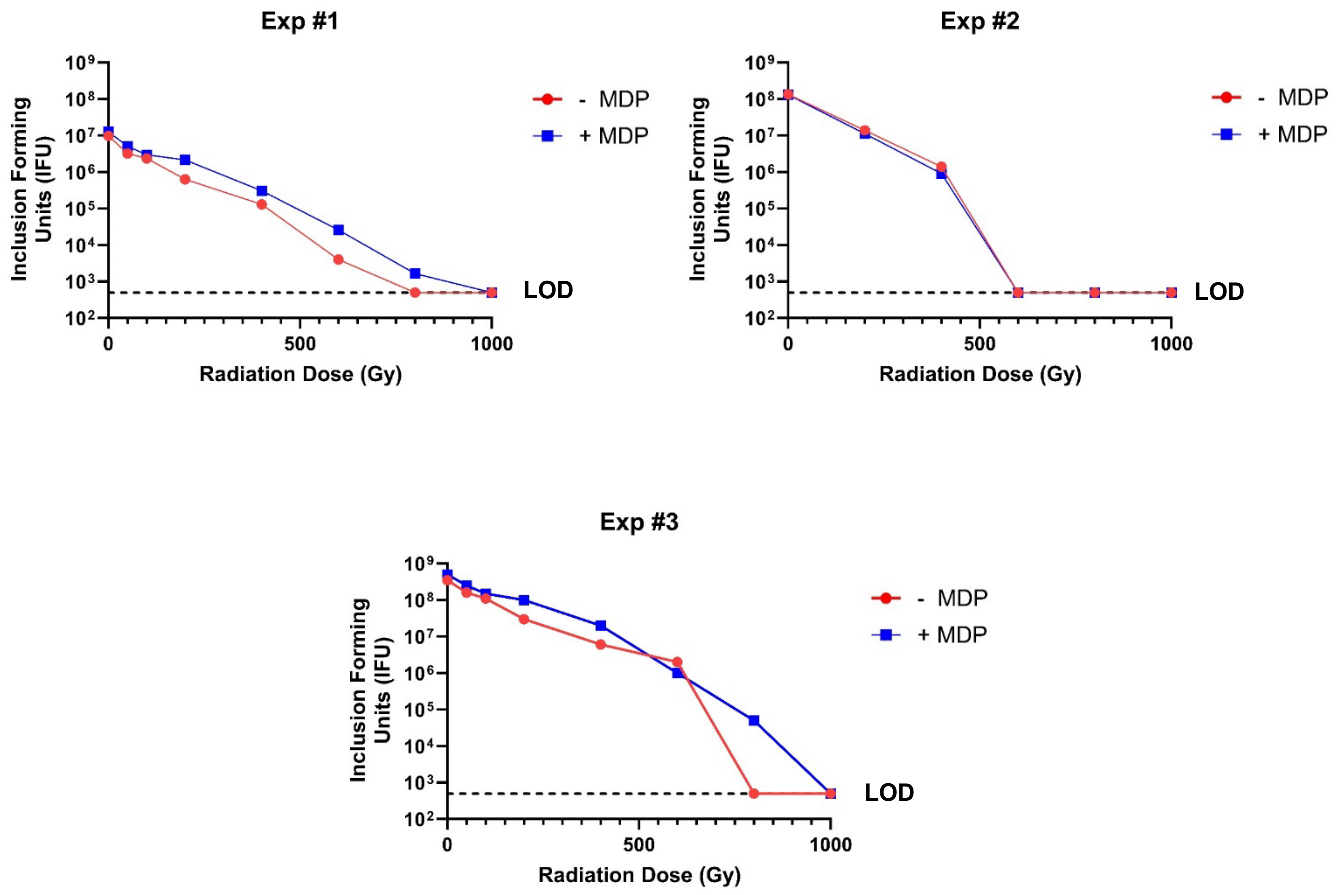

b

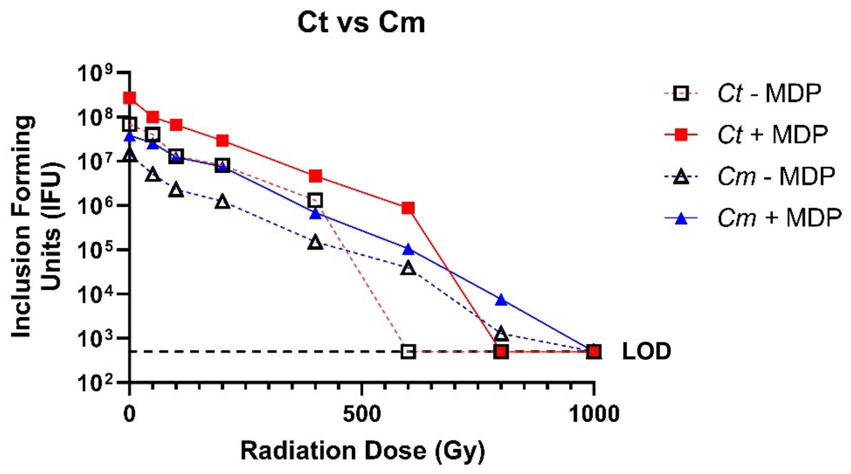

Supplementary Figure 1

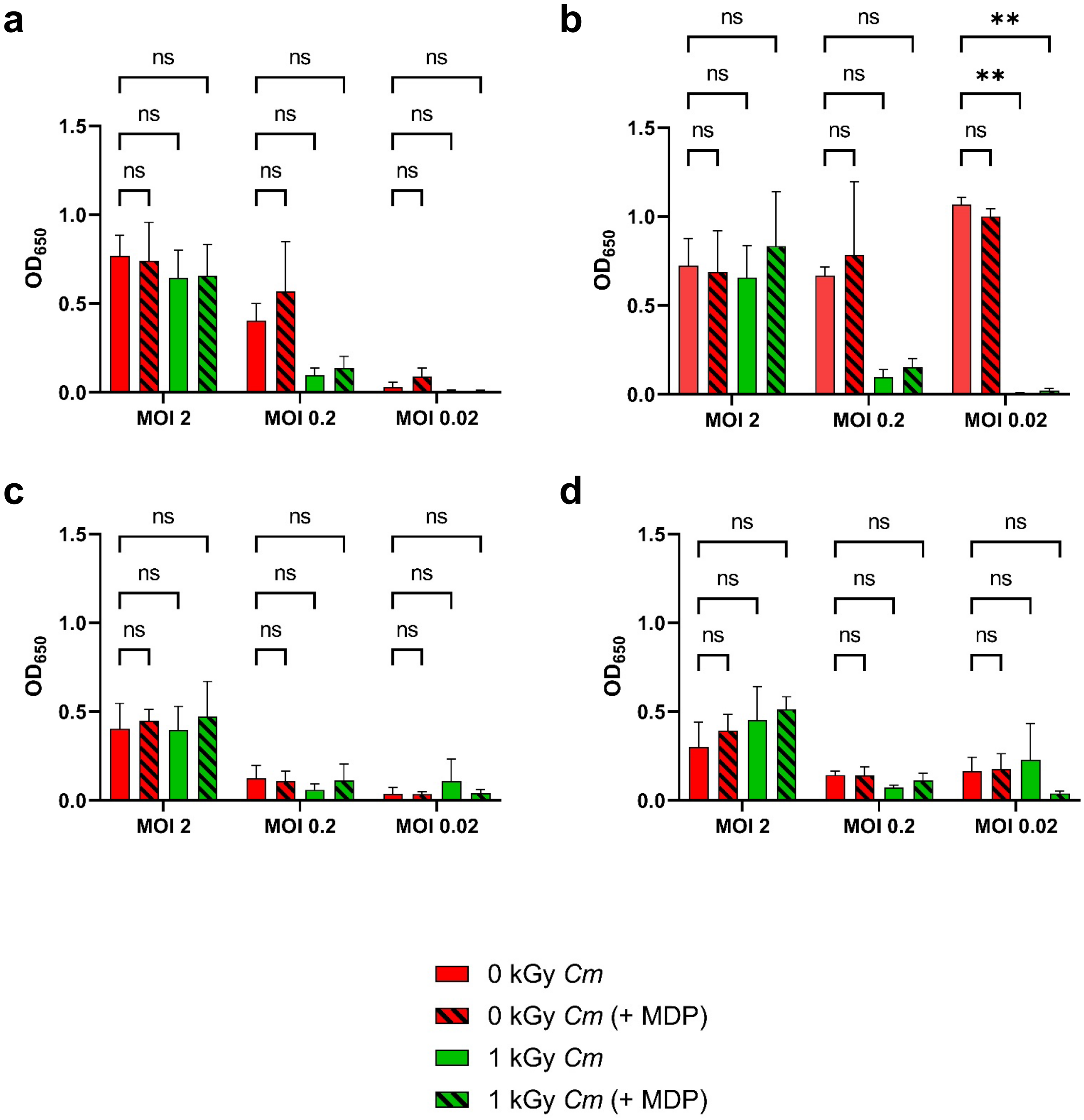

Supplementary Figure 2

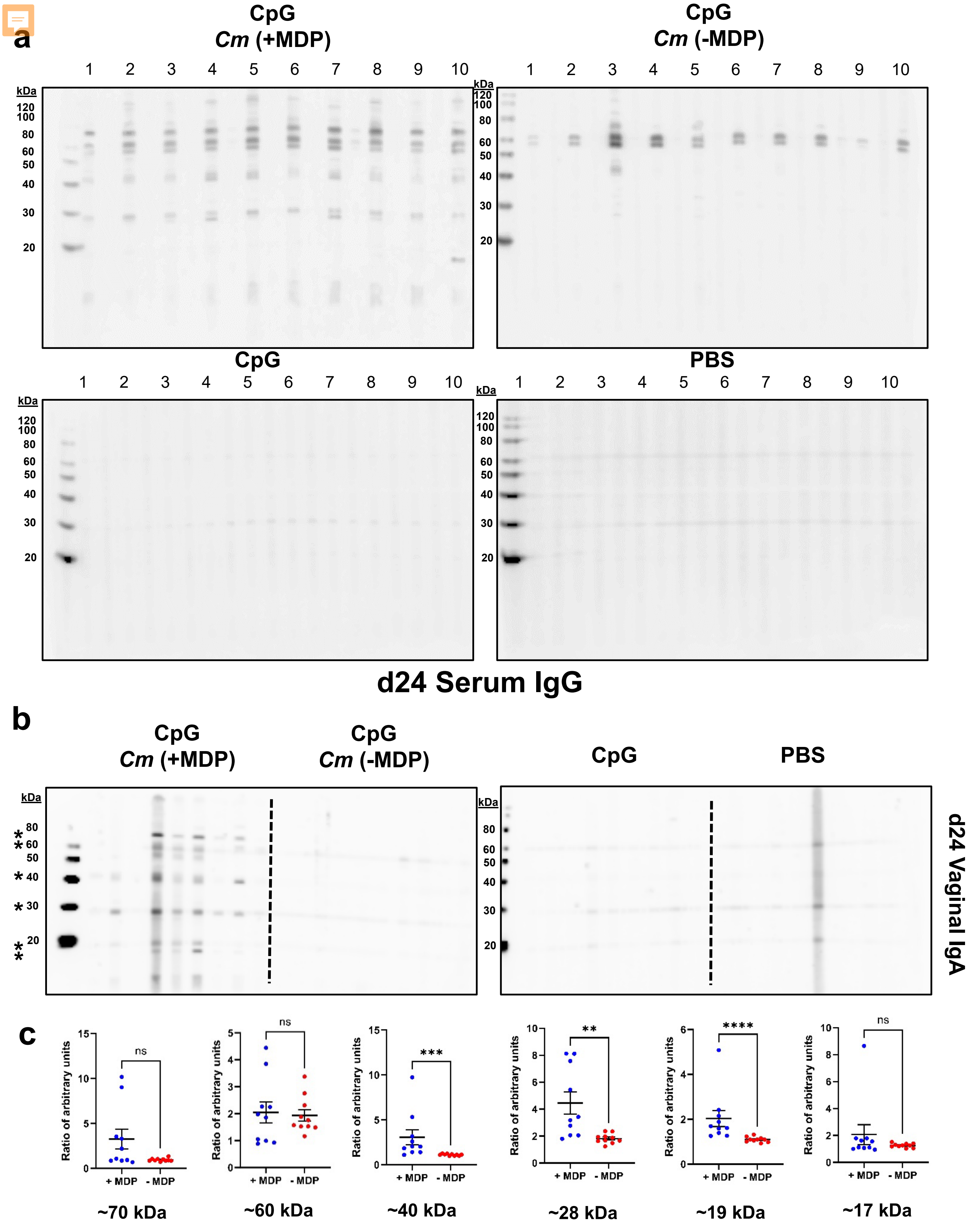

Supplementary Figure 3

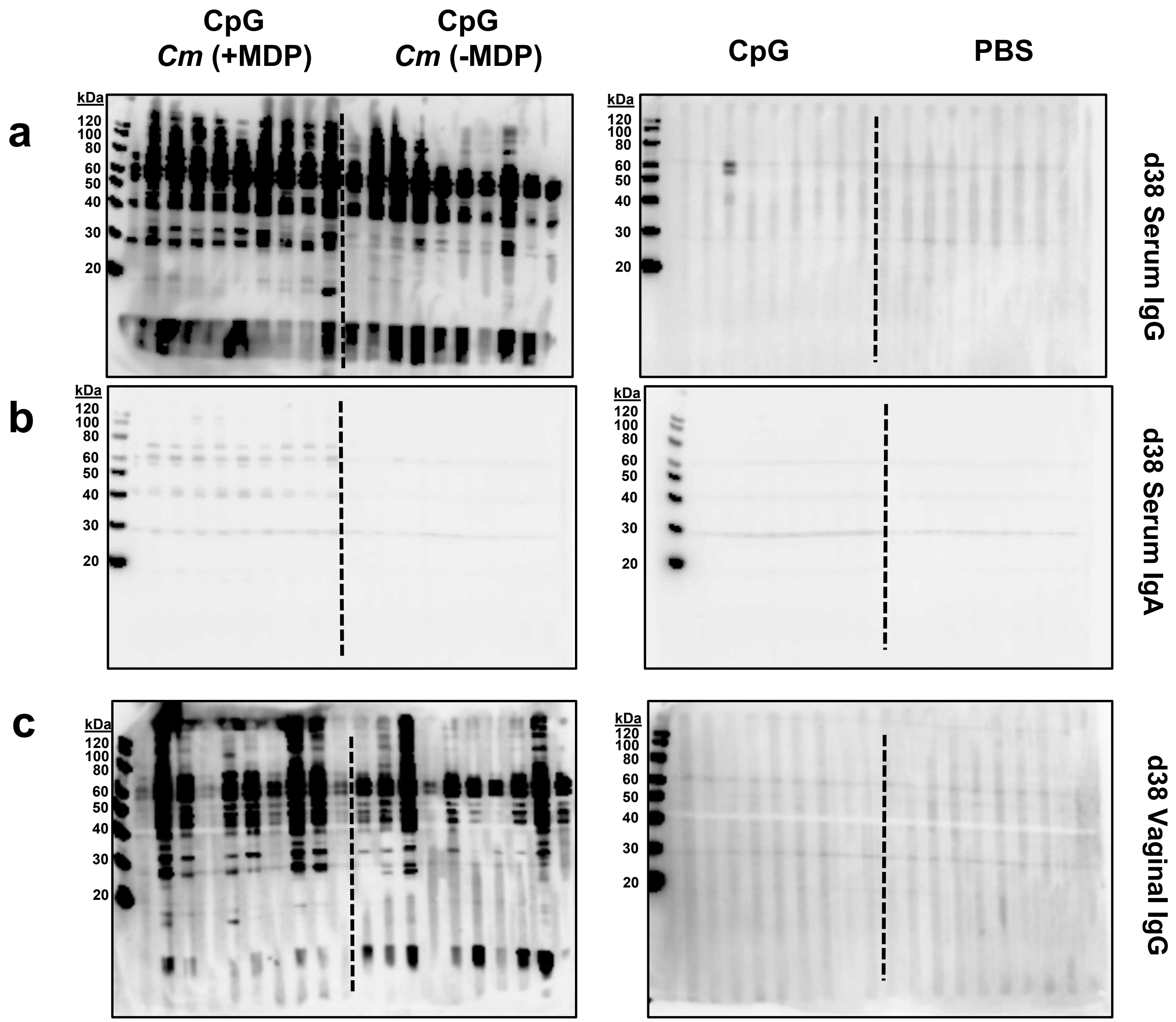

Supplementary Figure 4

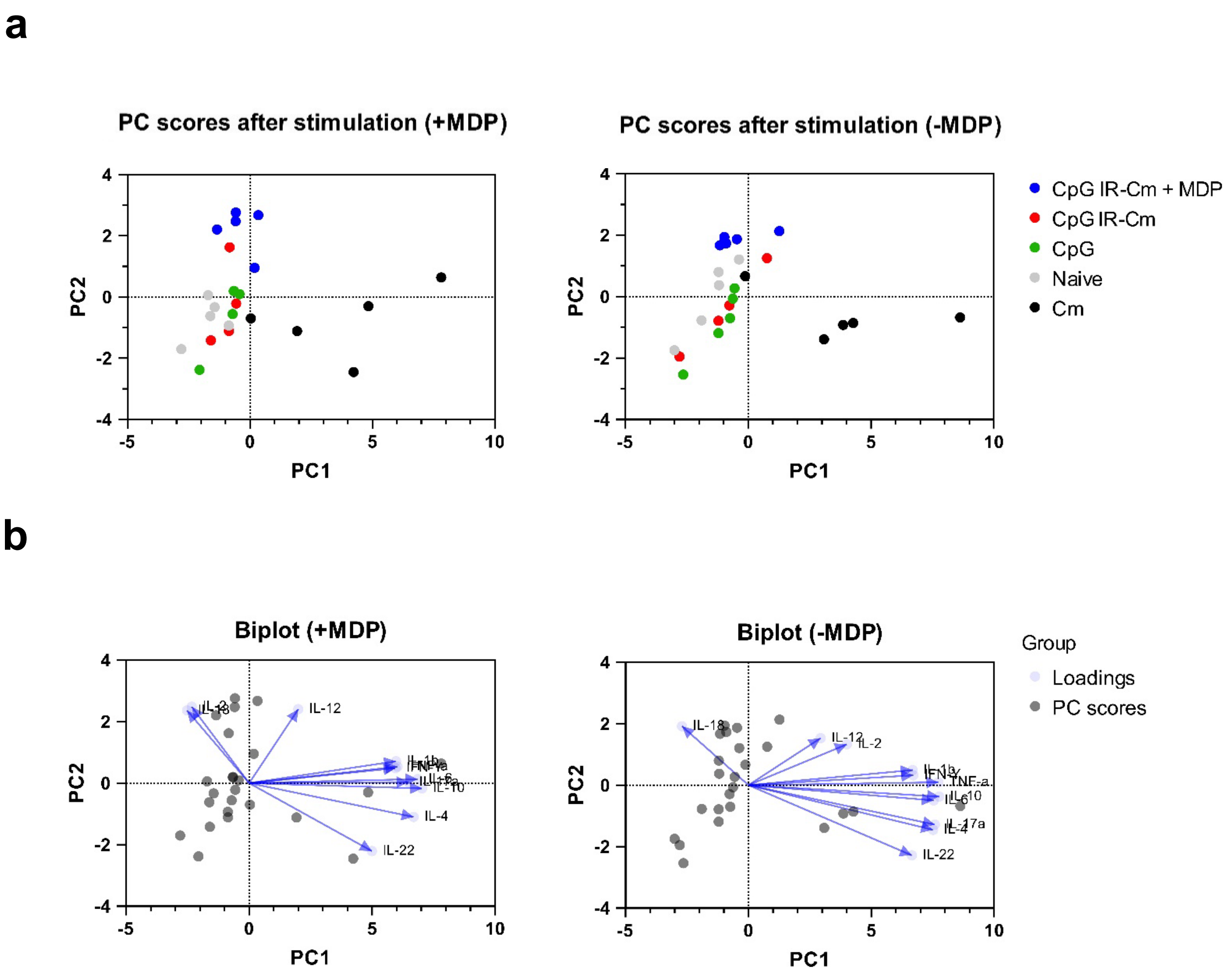

Supplementary Figure 5

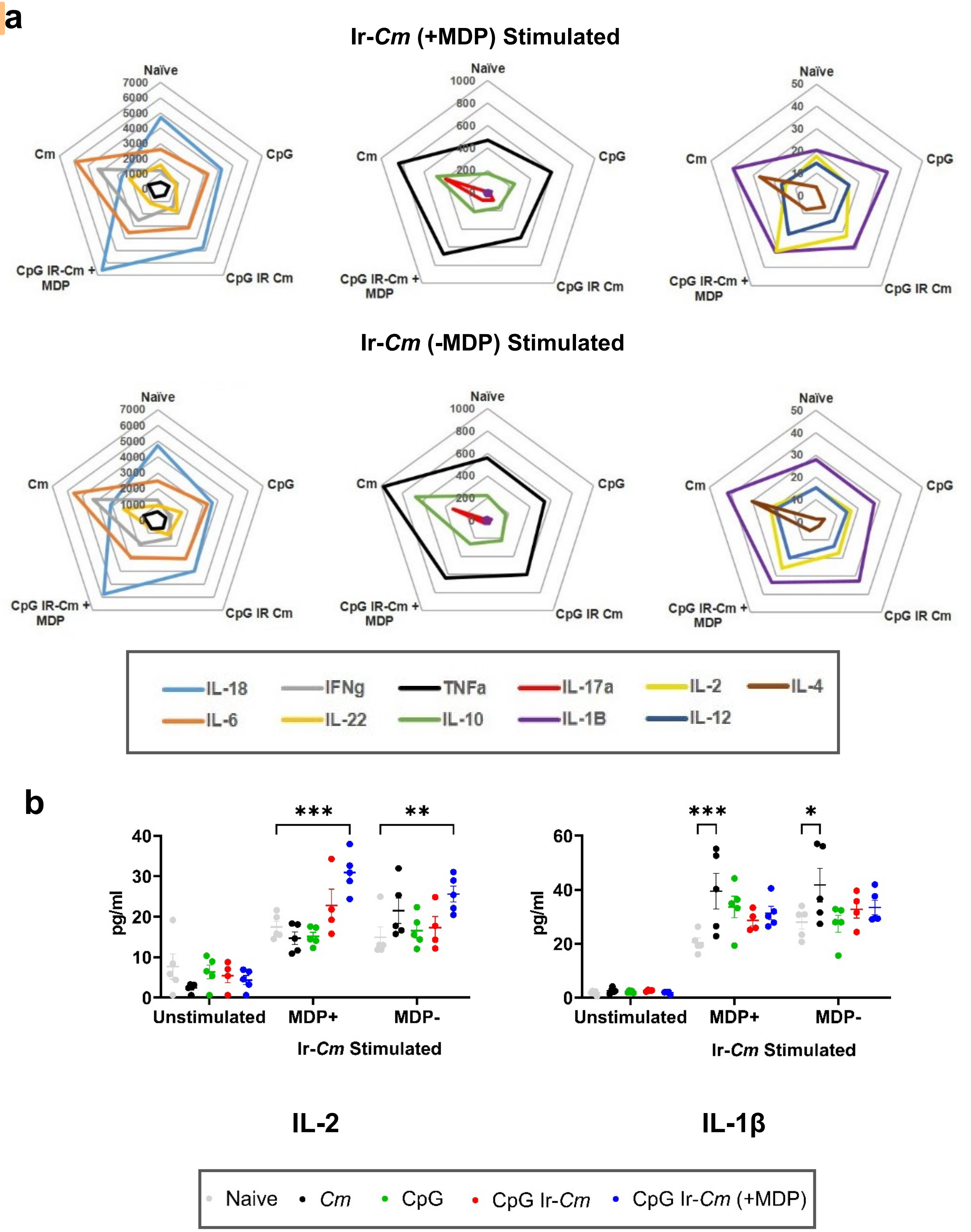

Supplementary Figure 6

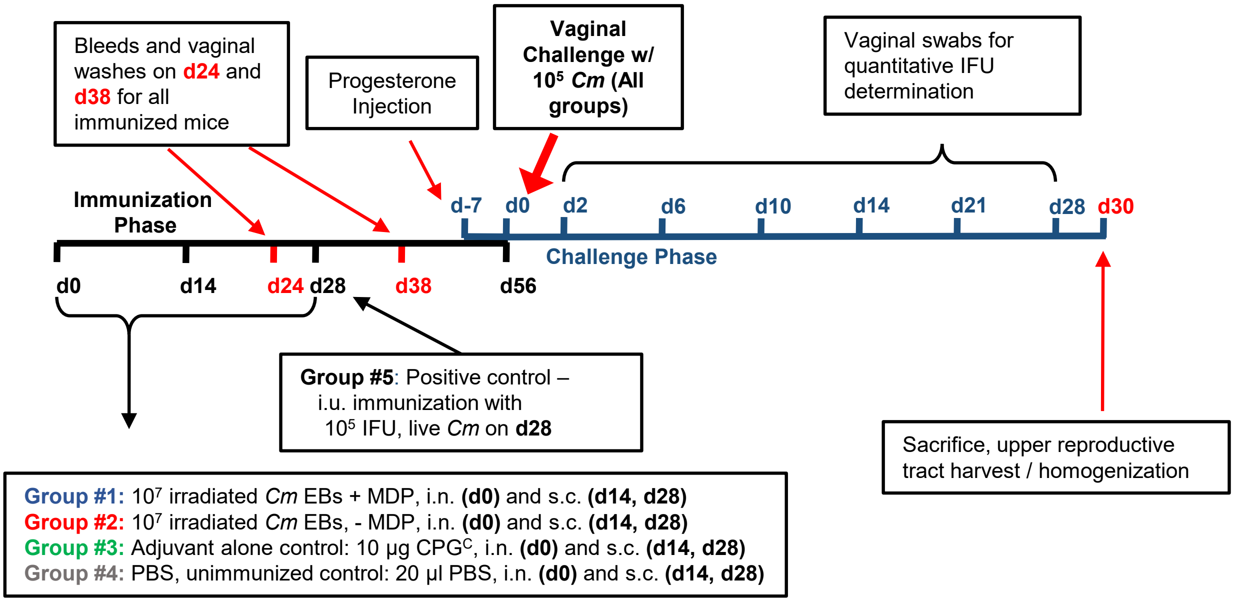

Supplementary Figure 7

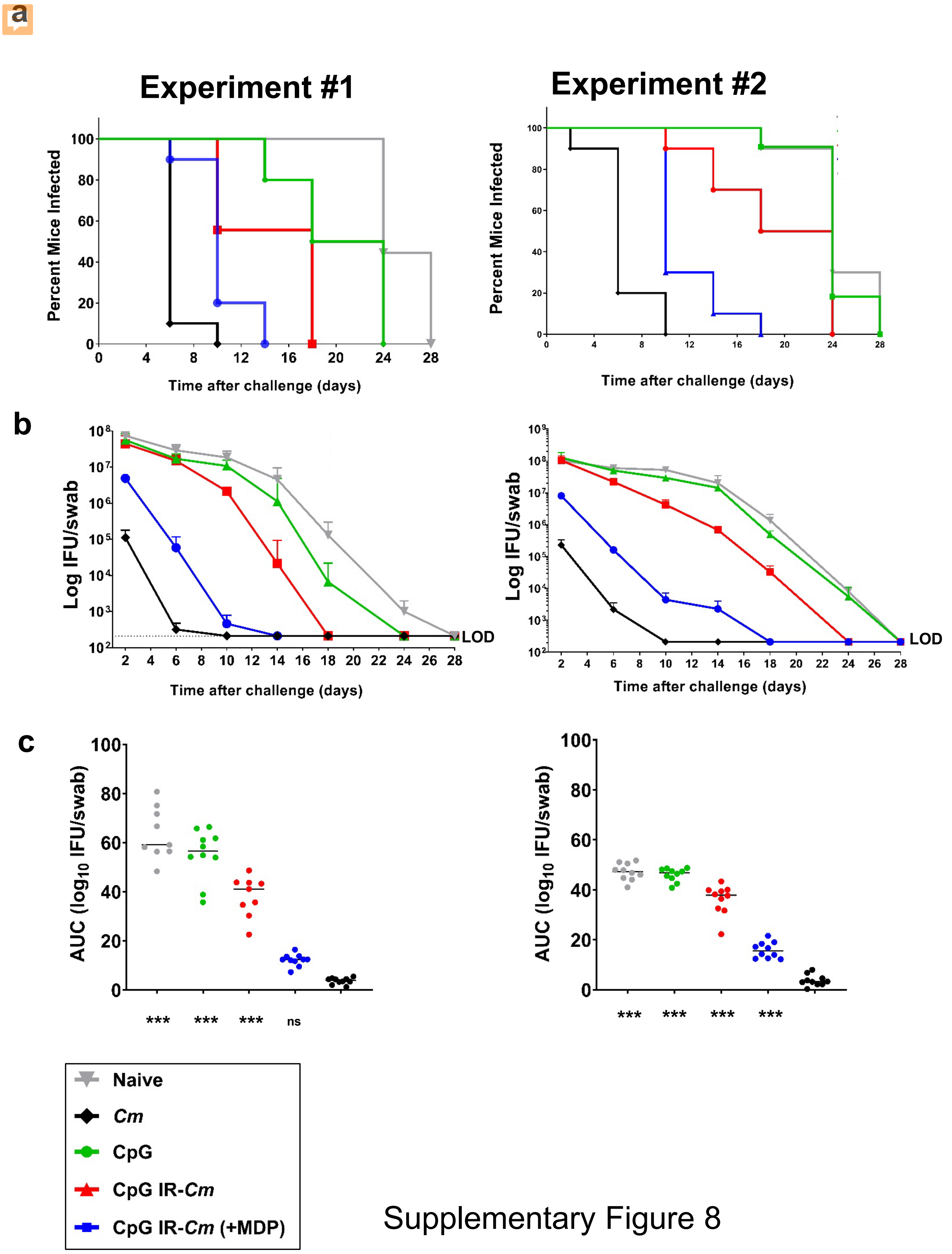

Supplementary Figure 8

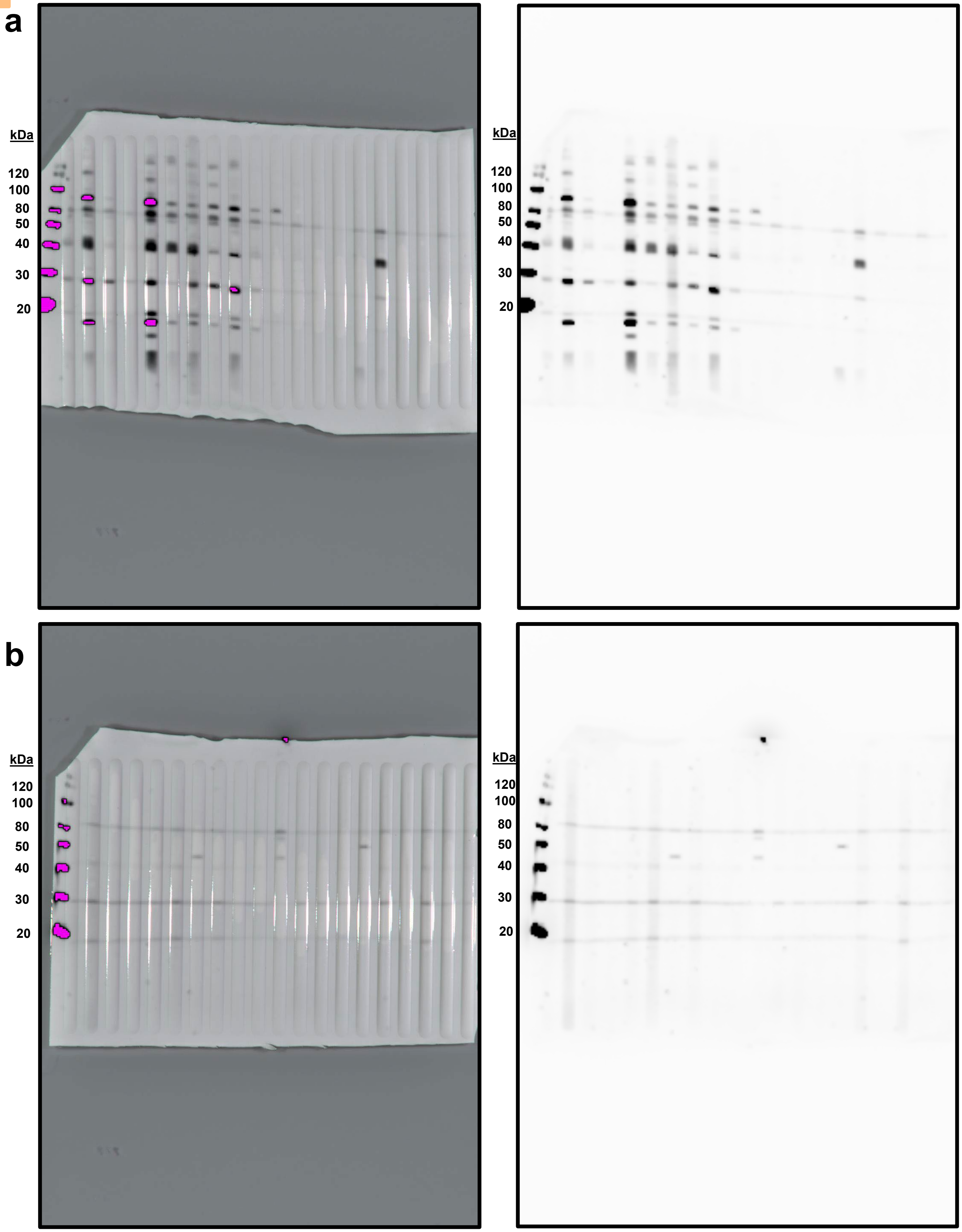

Supplementary Figure 9

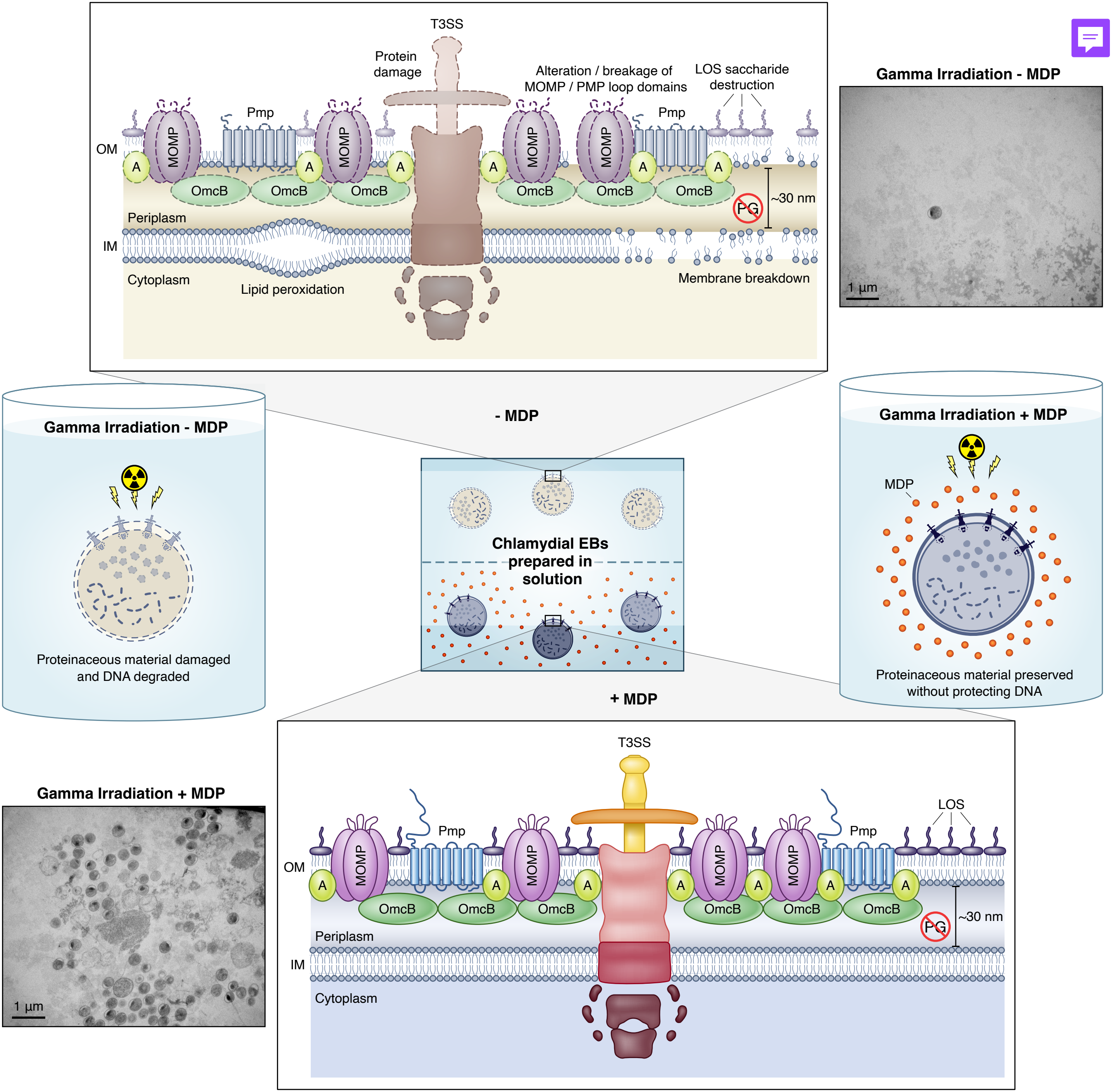
T3SS
Protein damage
Alteration / breakage of MOMP / PMP loop domains
LOS saccharide destruction
Pmp
MOMP
OM
A
OmcB
PG
~30 nm
Periplasm
IM
Cytoplasm
Lipid peroxidation
Membrane breakdown
Gamma Irradiation - MDP
1 µm
- MDP
Gamma Irradiation - MDP
Proteinaceous material damaged and DNA degraded
Chlamydial EBs prepared in solution
Gamma Irradiation + MDP
MDP
Proteinaceous material preserved without protecting DNA
+ MDP
Gamma Irradiation + MDP
1 µm
LOS

Supplemental Table 1. **Mantel-Cox tests for all groups presented in the % colonization study (Fig. 4a).**

	CpG IR-CM + MDP	CpG	CpG IR-CM	Naive	Cm
**CpG IR-CM + MDP**		<0.0001	0.0012	<0.0001	0.0004
**CpG**	<0.0001		0.0185	0.6269	<0.0001
**CpG IR-CM**	0.0012	0.0185		<0.0001	<0.0001
**Naive**	<0.0001	0.6269	<0.0001		<0.0001
**Cm**	0.0004	<0.0001	<0.0001	<0.0001	
